# Decontaminate Traces From Fluorescence Calcium Imaging Videos Using Targeted Non-negative Matrix Factorization

**DOI:** 10.3389/fnins.2021.797421

**Published:** 2022-01-21

**Authors:** Yijun Bao, Emily Redington, Agnim Agarwal, Yiyang Gong

**Affiliations:** ^1^Department of Biomedical Engineering, Duke University, Durham, NC, United States; ^2^North Carolina School of Science and Mathematics, Durham, NC, United States; ^3^Department of Neurobiology, Duke University, Durham, NC, United States

**Keywords:** neuroimaging, fluorescence calcium imaging, two-photon imaging, one-photon imaging, signal unmixing, decontamination, non-negative matrix factorization

## Abstract

Fluorescence microscopy and genetically encoded calcium indicators help understand brain function by recording large-scale *in vivo* videos in assorted animal models. Extracting the fluorescent transients that represent active periods of individual neurons is a key step when analyzing imaging videos. Non-specific calcium sources and background adjacent to segmented neurons contaminate the neurons’ temporal traces with false transients. We developed and characterized a novel method, temporal unmixing of calcium traces (TUnCaT), to quickly and accurately unmix the calcium signals of neighboring neurons and background. Our algorithm used background subtraction to remove the false transients caused by background fluctuations, and then applied targeted non-negative matrix factorization to remove the false transients caused by neighboring calcium sources. TUnCaT was more accurate than existing algorithms when processing multiple experimental and simulated datasets. TUnCaT’s speed was faster than or comparable to existing algorithms.

## Introduction

The brain has many neurons that coordinate their activity to support complex dynamics and behaviors. Neuroscientists often probe the relationship between the activity of neurons and animal behavior using recording techniques such as electrophysiology or optical microscopy. Electrophysiology can accurately quantify the action potentials and voltage activity of ensembles of neurons, but it is somewhat invasive and lacks dense sampling of all neurons in a small brain region. Optical microscopy (Helmchen and Denk, 2005; Grewe et al., 2010; Stringer et al., 2019; Wang et al., 2019; Wang et al., 2021) can densely record from many neurons over a large field of view simultaneously, chronically, and *in vivo* when imaging the activity reported by fluorescent genetically encoded calcium indicators (Akerboom et al., 2013; Chen et al., 2013; Dana et al., 2019; Inoue et al., 2019; Zhang et al., 2020). Because imaging movies are large and high-dimensional, neuroscientists typically extract information from individual neurons within these movie via a multi-step process, including registration, identifying active neurons, extracting calcium traces, and inferring neural spikes (Pachitariu et al., 2017; Giovannucci et al., 2019; Bao et al., 2021).

**FIGURE 1:**
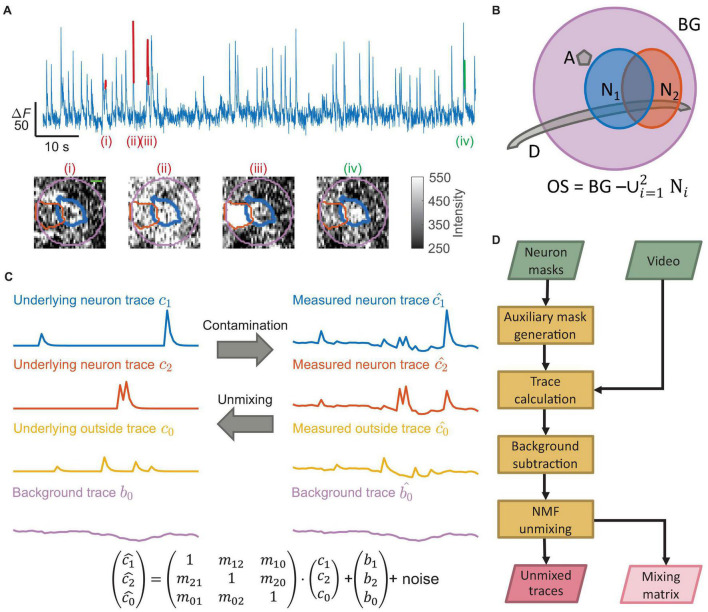
Neural signal contamination arises from neighboring cells, neural processes, or background. **(A)** An example temporal trace from one neuron showed multiple false transients. Four labeled transients (i)–(iv) show four examples with different origins, corresponding to the images below the trace. Each image is the corresponding spatial profile of fluorescence at the peak of each labeled transient. The colored contours in each image show the boundary of the segmented neuron (thick blue), the boundary of a neighboring neuron (orange), and the background region (purple) surrounding the segmented neuron (scale bar: 5 μm). Only transient (iv) was a true transient of the neuron (green), where the spatial profile of activation matched the neuron shape. The remaining transients were false transients (red) caused by contamination with spatial profiles not matching the neuron shape. Transient (i) came from a dendrite, transient (ii) came from background fluctuation, and transient (iii) came from an active neighboring neuron. **(B)** A schematic shows multiple typical contaminating sources around the neuron of interest (N_1_; blue); we show a neighboring neuron (N_2_; orange), an axon perpendicular to the imaging plane (A; gray), and a dendrite in the imaging plane (D; gray). In practice, multiple instances of each type of contaminating source may exist. The purple disk is the background region (BG) near the neuron. We defined the outside region (OS) as all pixels within the background region but not belonging to any neuron mask. **(C)** Matrix multiplication can represent the fluorescence contamination between regions. The mixture of the uncontaminated neuron traces (*c*_1_ and *c*_2_), the outside trace (*c*_0_), and the background trace (*b*_0_) generated the measured traces *c*_1_, *c*_2_, and *c*_0_. This work produced the uncontaminated traces from the measured traces. **(D)** A flow chart of our unmixing algorithm, TUnCaT. The inputs are the video and the neuron masks, and the outputs are the unmixed traces and the mixing matrix.

One important video processing step is the extraction of temporal fluorescence traces from neurons. The typical fluorescent protein calcium sensors respond to action potentials of excitatory neurons with a stereotypic transient temporal pattern, including a rapid rise and slow decay. While optical imaging has sufficient spatial resolution to resolve individual neurons, the recording technique still mixes signals from individual neurons with contaminating signals laterally or axially adjacent to those neurons; the contaminating signals originate from neighboring neurons, axons, dendrites, or bulk background (Figures 1A,B). This mixture in neuron traces may introduce false transients indistinguishable from true transients (Figures 1A,C). These false transients decrease the fidelity of identifying neural activity, and may even result in incorrect scientific conclusions about the neural dynamics or function (Gauthier et al., 2018). Although manually removing false transients is possible, it is prohibitively labor-intensive and slow for movies with many neurons spanning minutes or hours. Therefore, an accurate, automated trace decontamination algorithm is needed to obtain accurate neural activity traces.

Over the past decade, researchers have developed several algorithms to extract the underlying uncontaminated calcium traces from imaging videos. These algorithms mainly fall into two categories: three-dimensional spatiotemporal unmixing and one-dimensional temporal unmixing. Three-dimensional spatiotemporal unmixing algorithms identified active neurons and extracted their calcium traces concurrently (Mukamel et al., 2009; Maruyama et al., 2014; Pnevmatikakis et al., 2016; Pachitariu et al., 2017; Giovannucci et al., 2019). These methods modeled the fluorescence movie as a linear superposition of contributions from each neuron; each contribution was the tensor product of one neuron’s spatial footprint and the neuron’s temporal trace. The algorithms then found the optimal spatial and temporal decomposition by iteratively minimizing a cost function. Although extracting the spatial and temporal components of neurons simultaneously is convenient, current spatiotemporal unmixing algorithms have underperformed recent deep-learning-based spatial segmentation algorithms in speed and accuracy when assessing the quality of the identified neuron spatial footprints (Soltanian-Zadeh et al., 2019; Bao et al., 2021). Missing or inaccurate neuron spatial segmentations naturally led to missing or inaccurate neural activity detection.

Another category of unmixing algorithms used either manually curated or algorithm generated neuron masks and unmixed the one-dimensional traces only in the temporal domain. These methods first calculated sets of raw traces from sets of neurons and neighboring regions by averaging the fluorescence of all pixels in each region; the methods then unmixed the resulting traces to properly assign transients to each source region. Starting with very accurate spatial masks, these methods could improve the accuracy of calculating neural traces; accurate traces could then help improve the accuracy of determining the active periods of each neuron; accurate detection of the neural ensemble’s active periods eventually feeds into stronger biological conclusions about the dynamics and function of neural activity during animal behavior. The Allen Software Development Kit (Allen SDK) and Fast Image Signal Separation Analysis (FISSA) are two existing and leading temporal unmixing algorithms. The Allen SDK (de Vries et al., 2020) used frame-by-frame linear regression to reduce crosstalk contamination caused by overlapped pixels of neighboring neurons. However, their mixing model did not consider contaminations coming from small spatial scales near the neurons of interest, such as axons and dendrites. FISSA (Keemink et al., 2018) applied non-negative matrix factorization (NMF) to a matrix containing the trace from the neuron of interest and traces from adjoining but non-overlapped spatial regions. This algorithm recognized and removed false signals from the neuropil regions, but could inaccurately decontaminate signals from neurons that spanned multiple adjoining regions.

In this work, we developed a new algorithm, temporal unmixing of calcium traces (TUnCaT). TUnCaT decontaminated false contributions from all types of sources. TUnCaT combined the advantages of the Allen SDK and FISSA by applying background subtraction and NMF to a targeted set of traces from the neuron of interest, neighboring neurons, and the outside region containing axons and dendrites. It effectively and efficiently assigned false transients to contaminating sources and thus removed these transients from the trace of the neuron of interest. TUnCaT was more accurate than existing algorithms in experimental two-photon videos, simulated two-photon videos, and experimental one-photon videos; it was also faster than or comparable in speed to existing algorithms when processing these datasets.

## Materials and Methods

### Datasets and Characterization Scheme

We compared the performance of TUnCaT with the performance of other unmixing algorithms using three datasets: experimental two-photon videos from Allen Brain Observatory (ABO), simulated two-photon videos using Neural Anatomy and Optical Microscopy (NAOMi), and experimental one-photon videos.

#### Experimental Two-Photon Videos From Allen Brain Observatory

The ABO dataset we used included 10 videos used in our previous work (de Vries et al., 2020). These videos were recorded from 275 μm deep in the primary visual cortex (VISp) of 10 mice using two-photon microscopy; all mice expressed the GCaMP6f calcium sensor. The pixel size was 0.785 μm/pixel. Each video lasted about 13 min at a frame rate of 30 frames/s. We used our previous manual annotations (Soltanian-Zadeh et al., 2019) as the ground truth (GT) neuron markings, which were created for videos laterally cropped to 487 × 487 pixels; each video contained 235–386 GT neurons. To reduce the workload of manual transient labeling, we further cropped each frame to the center 200 × 200 pixels (157 μm × 157 μm); we cropped the masks to the center 200 × 200 pixels as well, and eliminated the masks with less than one-third of their areas in the cropped center region. Each video contained 46–67 remaining GT neurons, and the average neuron density was (2.3 ± 0.3) × 10^3^ neurons/mm^2^.

#### Simulated Two-Photon Videos Using Neural Anatomy and Optical Microscopy

We used the NAOMi simulation toolbox to simulate 10 two-photon imaging videos (Song et al., 2021). We used the available MATLAB code at https://codeocean.com/capsule/7031153/tree/v1 (version 1.1) (Charles et al., 2021) to implement the NAOMi simulation. We changed the indicator concentration from 200 to 10 μM to better match experimental conditions (Dana et al., 2014). We simulated a volume of 100 × 100 × 100 voxels, and the volume was 100 μm under the brain surface. The voxel size was 1 μm/pixel in each direction. The resulting videos had a lateral size of 90 × 90 pixels (90 μm × 90 μm) after removing edge pixels. We set the numerical aperture (NA) of the objective lens as 0.8, and the NA of the illumination light as 0.6. We set the average radius of each neuron as 5.9 pixels, and the minimum distance between any neurons as 12 pixels. We set the spiking rate as 0.05/s. For various noise parameters, we set mean measurement increase per photon (mu) as 10, electronics offset (mu0) as 0, variance increase per photon (sigma) as 23, and electronics base noise variance (sigma0) as a variable default at 2.7. We varied multiple parameters in simulation: recording frame rate, video length, number of neurons, laser power, electronics base noise variance, and GCaMP type. Our default parameter set specified the frame rate as 30 Hz, the simulated video length as 120 s, the number of neurons in the volume as 200, the laser power as 100 mW, the electronics base noise variance as 2.7, and the sensor as GCaMP6f. We set the number of dendrites in the volume as 20% of the number of neurons in the volume. The first 20 s of the simulation contained transient fluorescence, and were removed from consideration. We tested six types of calcium sensors: GCaMP6f (default), GCaMP6s, jGCaMP7f, jGCaMP7s, jGCaMP7b, and jGCaMP7c. The simulation parameters for GCaMP6s videos were the same as the default parameters for GCaMP6f videos. Some simulation parameters for GCaMP7 series sensors were different to accommodate the sensors’ slower kinetics. For these sensors, we set the frequency to 3 Hz, the simulated video length to 1100 s, the spiking rate to 0.01/s, and the initial transient period to 100 s.

Although NAOMi simulated many neurons in a volume, many of the simulated neurons were located out of the depth of focus and thus were not clearly visible at the principal imaging plane. We removed small or dim features at the simulation plane using the following steps. We binarized the spatial intensity profile of each neuron by using 0.2 times the maximum intensity of the spatial profile as the threshold. We calculated the area of each neuron as the number of pixels in the thresholded spatial profile, and removed neurons with areas smaller than half of the average neuron area. We calculated the integrated intensity over the spatial profile of each neuron, and removed neurons whose integrated intensity was lower than a threshold determined from the intensity distribution (∼60% neurons were removed in this step). We also removed neurons that did not have any action potentials after the 20 s transient period of the simulation (or 100 s for GCaMP7 series indicators). For each remaining neuron, we exported the thresholded spatial profile as the GT mask, and exported the clean trace as the GT trace. When using the default parameter set, each simulated video contained 18–55 GT neurons that met the above criteria, and the average neuron density was (4.3 ± 1.3) × 10^3^ neurons/mm^2^.

#### Experimental One-Photon Videos

All animal handling and imaging procedures were performed according to protocols approved by Duke Institutional Animal Care and Use Committee (protocol number A243-20-12). We recorded three one-photon imaging videos from the hippocampus cornu ammonis area 1 (CA1) region of mice expressing GCaMP6f. All experiments used male C57BL/6 mice kept in a standard day/night cycle. Mice were 8–9 weeks old at the time of the first surgery. During the first surgery, we injected CA1 with 500 nL of the viral vector AAV2/1-CaMKIIα-GCaMP6f (6.5 × 10^13^ vg/mL) at 100 nL/min using a pulled glass pipette. After at least 2 weeks, we aspirated the overlying cortical tissue and implanted a viewing window above the injection site. The injection and optical window implantation were similar to previous work (Mohammed et al., 2016). Mice were at most 12 weeks old at the time of the last implantation surgery. Following surgery, mice housing was communal, with up to five mice in a cage.

We imaged mice with a custom one-photon microscope, a 10 × /0.3 NA objective (Plan Fluor; Nikon), a dual-channel filter set (59009; Chroma), 505 nm LED illumination (M505L4; Thorlabs), and a scientific CMOS camera (Flash4v2; Hamamatsu). The final magnification was 7.5×, and the field-of-view was 1.4 × 0.85 mm. We used excitation powers of 0.18–0.33 mW/mm^2^, depending on the expression level of GCaMP6f. Each video lasted about 35 min at a frame rate of 20 frames/s. We removed motion artifacts by registering the videos (Guizar-Sicairos et al., 2008; Pnevmatikakis and Giovannucci, 2017). We spatially binned each video by a factor of two. After spatial binning, the pixel size was 3.5 μm/pixel. We segmented active neurons using CNMF-E (Zhou et al., 2018) on videos filtered with a bandpass filter (lower FWHM = 2 μm, upper FWHM = 160 μm); we then manually selected true masks from the output of CNMF-E. We spatiotemporally cropped three non-overlapped sub-videos from each video with a size of 50 pixels × 50 pixels × 6,000 frames (175 μm × 175 μm × 6,000 frames). We matched these crops with spatial crops of the neuron masks, and eliminated the masks with less than one-third of their areas in the cropped regions. This process generated nine independent sub-videos; each video contained 25–47 GT neurons, and the average neuron density was (1.3 ± 0.3) × 10^3^ neurons/mm^2^.

#### Generating Signal-to-Noise Ratio Videos

To better identify transients of neurons, we generated a signal-to-noise ratio (SNR) video for each raw imaging video (Bao et al., 2021). We first removed spatially large background fluctuation by applying a spatial homomorphic filter (Oppenheim et al., 1968), and then enhanced the SNR of calcium transient waveforms by applying a temporal matched filter tailored for shot noise (Wilt et al., 2013). Finally, we highlighted active transients and de-emphasized inactive periods by whitening the fluorescence time series of each pixel: we subtracted each time series by its baseline estimated from the median over time, and divided the difference by the noise estimated by the quantile-based standard deviation (Bao et al., 2021).

### Hardware and Speed Analysis

All algorithms were evaluated on a single computer (Windows 10, AMD 1920X CPU, 128 GB RAM).

### Temporal Unmixing of Calcium Traces

The workflow of TUnCaT has four stages: auxiliary mask generation, trace calculation, background subtraction, and NMF unmixing.

#### Auxiliary Mask Generation, Trace Calculation, and Background Subtraction

For each neuron, we generated a background mask and an outside mask as auxiliary masks. We defined the background mask as a disk centered at the neuron centroid, and the radius was 2.5 times the radius of a circle with an area equal to the average area of all neuron masks of the video. We defined neighboring neurons as the neurons whose centroids fell inside the background mask. We defined the outside region as all pixels within the background mask but not belonging to any neuron mask (including the neuron of interest and the neighboring neurons); if the area of the outside mask was smaller than half of the average neuron area, we recursively increased the radius of the outside mask by one pixel until the area was larger than that threshold. For each neuron, we generated a set of three traces: we calculated the raw trace of each neuron and the raw trace of the outside region by averaging the intensities of all pixels inside each corresponding mask on each frame; we calculated the raw background trace as the median of all pixels within the background mask on each frame. We then converted each set of three traces into a set of two background-subtracted traces, where the background trace of each set was subtracted from the neuron and outside traces within the same set. We finally grouped the background-subtracted traces of the neuron (${\tilde{c}}_{1}$) and outside region (${\tilde{c}}_{0}$) associated with each neuron of interest with the background-subtracted neuron traces of neighboring neurons (${\tilde{c}}_{k}$, *k* ≥ 2 if any neighboring neurons existed) as *F*_meas_,


$$F_{\text{meas}}=\left(\begin{array}{c} {\tilde{c}}_{1}\\ {\tilde{c}}_{2}\\ \ldots \\ {\tilde{c}}_{k}\\ {\tilde{c}}_{0} \end{array}\right),$$

the input to NMF. We performed the above calculation for multiple neurons in parallel over multiple central processing unit (CPU) cores using the multiprocessing or numba module to improve the speed.

#### Non-negative Matrix Factorization Unmixing

Non-negative matrix factorization is a widely used unsupervised learning algorithm that extracts non-negative features from input data. We applied NMF to the pre-processed traces because each trace satisfied the non-negativity constraint. *F*_meas_ and the underlying calcium activity from separated components without contaminations (*F*_sep_) were related by a matrix multiplication


$$F_{\text{meas}}=M\,F_{\text{sep}},$$

$$\left(\begin{array}{c} {\tilde{c}}_{1}\\ {\tilde{c}}_{2}\\ \ldots \\ {\tilde{c}}_{k}\\ {\tilde{c}}_{0} \end{array}\right)=\left(\begin{array}{ccccc} m_{11} & m_{12} & \ldots & m_{1\,k} & m_{10}\\ m_{21} & m_{22} & \ldots & m_{2\,k} & m_{20}\\ \ldots & \ldots & \ldots & \ldots & \ldots \\ m_{k\,1} & m_{k\,2} & \ldots & m_{k\,k} & m_{k\,0}\\ m_{01} & m_{02} & \ldots & m_{0\,k} & m_{00} \end{array}\right)\cdot \left(\begin{array}{c} c_{1}\\ c_{2}\\ \ldots \\ c_{k}\\ c_{0} \end{array}\right).$$

Here, *c_i* is the uncontaminated trace associated with ${\tilde{c}}_{i}$. The square mixing matrix, *M*, had off-diagonal element *m*_*ij*_ (*i* ≠ *j*) representing the contaminating contribution of the component *j* to the measured trace *i*; diagonal elements *m*_*ii*_ were defined as 1. NMF estimated *M* and *F*_sep_ concurrently from *F*_meas_, while requiring all elements in *M* and *F*_sep_ to be non-negative.

Because our set of input signals were tied to physical sources, the dimensions of *F*_sep_ and *F*_meas_ were the same, and we did not use NMF for dimensionality reduction. Regularization precluded trivial solutions such as *F*_sep_ = *F*_meas_ and *M* being the identity matrix. We solved the NMF problem iteratively by minimizing the cost function


$$E=\frac{1}{2}\,{||F_{\text{meas}}-M\cdot F_{\text{sep}}||}_{{\text{Fro}}^{2}}+\alpha \cdot \text{reg}\,\left(M,F_{\text{sep}}\right),$$

where ${||A||}_{{\text{Fro}}^{2}}=\frac{1}{2}\,\sum_{i,j}A_{i\,j}^{2}$ was the Frobenius norm of a matrix. The first term was the residual term quantifying the difference between the decomposition and the input matrix. The constant α ≥ 0 was the regularization parameter controlling the relative weight of the regularization term. The second term was the regularization function penalizing large norms on the decomposed matrices,


$$\text{reg}\,\left(M,F_{\text{sep}}\right)=l_{1}\,{||M||}_{1}+l_{1}\,{||F_{\text{sep}}||}_{1}+\left(1-l_{1}\right)\,{||M||}_{{\text{Fro}}^{2}}$$

$$+\left(1-l_{1}\right)\,{||F_{\text{sep}}||}_{{\text{Fro}}^{2}},$$

where ${||A||}_{1}=\frac{1}{2}\,\sum_{i,j}\left|A_{i\,j}\right|$ was the L_1_ norm of a matrix, and *l*_1_ was the ratio controlling the relative weight of the two types of norms in this function (0 ≤ *l*_1_ ≤ 1, and we used *l*_1_ = 0.5 throughout the work).

We normalized the input traces in *F*_meas_ by the quantile-base standard deviation (Bao et al., 2021) of the first row in *F*_meas_, so that the trace of the neuron of interest had a unity quantile-base standard deviation. We then subtracted the resulting matrix by its minimum value so that all the elements were non-negative. We used the NMF implementation in the scikit-learn toolbox (Pedregosa et al., 2011) with the following options and parameters: we chose the coordinate descent solver to iteratively solve the NMF problem. We initialized the decomposition using non-negative double singular value decomposition and replaced zeros with small random values. We stopped the iteration process after 20,000 iterations or until the tolerance was smaller than 10^–4^.

Because the order of the traces in *F*_sep_ was not deterministically matched to the input traces after NMF, we iteratively matched the traces in *F*_sep_ according to their relative contributions to the corresponding traces in *F*_meas_. The following five-step process ensured that the contribution of the trace *c*_*i*_ in *F*_sep_ to the trace ${\tilde{c}}_{i}$ in *F*_meas_ was larger than the contribution to any other trace ${\tilde{c}}_{j}$ in *F*_meas_ (*i* ≠ *j*).

(1) We normalized each column of *M* so that the sum of each normalized column was unity, but maintained the *MF*_sep_ product: we divided the *i*^th^ column of *M* by the sum of that column, and multiplied the *i*^th^ row of *F*_sep_ (*c*_*i*_) by the same number.
(2) We initialized two helper matrices *M*_0_ and *P*: *M*_0_ was initialized to *M*, and *P* was initialized to a matrix of zeros with the dimensions of *M*.
(3) We iteratively updated *M*_0_ and *P*. In each iteration, we located the largest element of *M*_0_ as *m*_0(_*_*i,j*_*_)_. Then we set *p*_*ij*_ = 1, set the elements in the *i*^th^ row or the *j*^th^ column of *M*_0_ as zeros, and normalized each column of *M*_0_ again if that column was not identically zero. We iterated this process until all the elements of *M*_0_ were zeros.
(4) We substituted *M* by the column-permuted matrix *MP^T^*, where the superscript T indicated transpose; we substituted *F*_sep_ by the row-permuted matrix *PF*_sep_.
(5) We normalized the diagonal of *M* to unity by dividing the *i*^th^ column of the permuted *M* by its diagonal element *m*_*ii*_ and multiplying the *i*^th^ row of the permuted *F*_sep_ by the same *m*_*ii*_.

We showed an example matching process in Supplementary Table 1. Finally, we reversed the normalization procedure before matrix factorization by multiplying each row in *F*_sep_ with the quantile-base standard deviation of the first row in *F*_meas_ to retain the correct amplitudes of the traces, and we aligned the medians of the unmixed traces with the medians of the input traces. We performed NMF unmixing for multiple neurons in parallel over multiple CPU cores using the multiprocessing module to improve the speed.

#### Floating α Strategy

Because all the input traces originate from masks with physical meaning, we required *F*_sep_ to have the same number of non-trivial signals as *F*_meas_; correspondingly, we required *M* to be a square matrix with all non-trivial columns. However, at large values of α, over-regularization often generated identically zero output traces in *F*_sep_. Even if such identically zero traces were not directly assigned to the neuron of interest, the zero traces could still indirectly cause the trace from the neuron of interest to improperly retain false transients. In these instances, we made α an internal parameter that automatically adjusted without further cross-validation optimization; we recursively divided α by 2 and repeated the NMF process until none of the traces in *F*_sep_ were identically zero. Therefore, the final α may differ from the initial α for each neuron. When using this floating α strategy, the initial α was the input used in the algorithm and was the same for all neurons in a test video. This was also the value we reported throughout the paper when it was obtained via cross-validation. Alternatively, a fixed α strategy applied only one round of NMF per neuron using the initial α value uniformly for all neurons in a test video.

#### Temporal Downsampling

We attempted to reduce computation time by determining the mixing matrix from a set of downsampled traces. We hypothesized that the mixing matrix produced by NMF would be relatively independent of the sampling rate. At the NMF stage, we temporally downsampled the input matrix *F*_meas_ at constant intervals throughout the movie, and calculated the corresponding decomposed mixing matrix, *M*′, for the downsampled input. We then estimated the output signals *F*_sep_ at the original temporal resolution using *M*′ and *F*_meas_, where *F*_meas_ was also at the original temporal resolution.

### Evaluation of Unmixing Accuracy

We first identified transients from the unmixed traces from each algorithm, and compared them with GT transients. The GT transients of experimental videos were manually labeled, while the GT transients of simulated videos were automatically calculated from GT traces. The matching between the algorithm-detected transients and the GT transients quantified using the *F*_1_ score represented the accuracy of the unmixing algorithms.

#### Transient Detection in Experimental Videos

Our transient detection algorithm was based on SNR thresholding. If the traces were calculated from raw videos, we first enhanced the SNR of the traces by applying the same temporal filtering technique as the temporal filtering step used in generating SNR videos; if the traces were calculated from SNR videos, we did not apply additional temporal filtering. After we enhanced the traces, we normalized each trace to an SNR trace by subtracting the estimated baseline and then dividing by the estimated noise; we estimated the baseline using the peak of the kernel smoothing density (Mitani and Komiyama, 2018), and estimated the noise using the high-frequency range of the power spectral density (Pnevmatikakis et al., 2016). We considered transients that satisfied two requirements: the transient should be sufficiently higher than the baseline, and the transient should have a prominent peak. We considered the neuron as potentially active on the frames when the SNR trace was higher than a threshold, *th*_SNR_, which we parameterized during cross-validation. We then grouped consecutive active frames into potentially active periods. We also screened for prominent peaks with a prominence of at least *th*_SNR_/3 (Mathworks, 2021). If a potentially active period determined in SNR thresholding contained exactly one prominent peak, we recorded this potentially active period as a transient. We defined the start of a transient as the first frame that the trace was above *th*_SNR_, and defined the end of a transient as the last frame that the trace was above *th*_SNR_. We discarded potentially active periods that did not contain any prominent peaks. If a potentially active period contained multiple prominent peaks, we split this active period into multiple transients by using the local minima between the prominent peaks as the endpoints. We defined the frame before a boundary local minimum as the end of the preceding transient, and we defined the frame after a boundary local minimum as the start of the following transient. We saved the start and end times of each transient for both manual labeling and accuracy evaluation.

#### Manual Labeling in Experimental Videos

We manually labeled true transients from all the experimental two-photon and one-photon videos used in this paper to produce the set of GT transients. We developed a graphical user interface to assist the manual labeling process. We first used the same transient detection algorithm described above to detect all potential transients (both true and false) of the traces. We then manually labeled each of these potential transients with aid from two visualizations: we played a short video from 20 frames before the start of the transient to 20 frames after the end of the transient, and we displayed the mean image over the transient duration. We overlaid the contours of the neuron of interest and neighboring neurons on top of each visualization using different colors. A human labeler classified each transient as true or false by comparing the neuron contours to the fluorescence footprint of the mean image or of the movie during the transient; qualitative high spatial matching between the contour of the neuron of interest and the fluorescence footprint during these peak intensity frames suggested that this potential transient was a true transient of the neuron of interest.

#### Transient Calculation From Simulated Ground Truth Traces

For simulated videos, we generated GT transients from the GT traces exported by NAOMi. We first estimated the size of fluorescence transients from each neuron in three steps. First, we applied the same temporal filtering to the GT traces as with experimental traces. Second, we screened for peaks with a prominence of at least the GT trace’s standard deviation. Third, because the simulation produced transients of nearly identical heights for all transients corresponding to individual action potentials, we calculated the estimated average height of a single transient of that neuron using the peak of the kernel smoothing density (Mitani and Komiyama, 2018) of all the peak heights. We then used half of this estimated average peak height as the activity threshold to determine the potentially active periods of the neuron. We also used half of the estimated average peak height as the minimum prominence of transient peaks to determine prominent peaks. We next followed the same procedures as used with experimental traces to process the potentially active periods and prominent peaks into GT transients.

#### Evaluation Metrics

We evaluated all unmixing methods by quantifying the matches between the algorithm-detected transients and the GT transients. We defined the distance of a pair of algorithm-detected transient and GT transient as the negative of the number of their overlapped frames, and thus generated a distance matrix between two sets of transients. Next, we applied the Hungarian algorithm to solve the linear assignment problem using the above distance matrix, and denoted the matched transients whose distances were non-zero as true positive transients. We defined the ratio of the number of true positive transients to the number of algorithm-detected transients in the video as the precision, and defined the ratio of the number of true positive transients to the number of GT transients in the video as the recall. We finally defined an *F*_1_ score that combined recall and precision as


$$F_{1}=2/\left({\text{Recall}}^{-1}+{\text{Precision}}^{-1}\right).$$

A higher *F*_1_ score indicated more accurate transient detections, and more accurately detected transients meant more accurate unmixing.

#### Cross-Validation

We optimized *th*_SNR_ and α to maximize our algorithm’s transient detection accuracy. We used *n*-round leave-one-out cross-validation to optimize these two parameters, where *n* = 10 videos for the experimental and simulated two-photon datasets, and *n* = 9 for the experimental one-photon dataset. In each round of cross-validation, we chose one video as the test video, and the remaining *n*−1 videos as the training videos. We used a grid search to find the *th*_SNR_ and α that maximized the mean *F*_1_ score on all the training videos. We then used the optimized parameters to detect transients from the test video, and evaluated the unmixing accuracy of these transients. We used each video of the dataset as the test video exactly once, and used the mean *F*_1_ score over all the test videos as the final metric that quantified the unmixing accuracy of each dataset. This cross-validation strategy also applied to FISSA. Because constrained non-negative matrix factorization (CNMF) and the Allen SDK did not use α, we used cross-validation to optimize only *th*_SNR_ when testing these two algorithms.

### Other Unmixing Algorithms

We compared TUnCaT with three other unmixing algorithms: FISSA (Keemink et al., 2018), CNMF (Pnevmatikakis et al., 2016; Giovannucci et al., 2019), and the Allen SDK (Allen Institute, 2019; de Vries et al., 2020).

#### FISSA

We used the available Python code at https://github.com/rochefort-lab/fissa (version 0.7.2) to implement the algorithm of FISSA. We optimized the regularization parameter α using cross-validation, and set all other parameters as default.

#### Constrained Non-negative Matrix Factorization

We used the available Python code of CaImAn at https://github.com/flatironinstitute/CaImAn (version 1.6.4) to implement the algorithm of CNMF. In principle, CNMF is a spatiotemporal unmixing method that identified active neurons and extracted their calcium traces concurrently. Nevertheless, the CaImAn package provided a module that seeded CNMF with a given set of neuron masks, so this module can work as a temporal unmixing method that exported only the temporal traces of known neurons. We used this module as a fair comparison to our temporal unmixing method. We set the autoregressive order as 1, and set the number of global background components as 2. CNMF exported a noiseless unmixed trace and a residual trace for each neuron; we perform the SNR analysis of CNMF on the sum of these two traces (Giovannucci et al., 2019), because the sum considered the noise of the system. Occasionally, CNMF produced fewer output traces than the number of input neurons; for the neurons without matching output traces, we set their output traces as identically zero, and detected no transients from those traces.

#### Allen SDK

We used the available Python code at https://github.com/AllenInstitute/AllenSDK (version 2.7.0) to implement the algorithm of the Allen SDK. We used the function *roi_masks.calculate_roi_and_neuropil_traces* to calculate the raw traces of neurons (*F*_*M*0_) and the traces of neuropils (*F*_*N*_). We used the function *demixer.demix_time_dep_masks* to unmix the traces of all neurons (*F*_*M*0_) simultaneously via frame-by-frame linear regression, which resulted in the unmixed traces of the neurons (*F*_*M*_). We used the function *r_neuropil.estimate_contamination_ratios* to estimate the neuropil contamination ratio (*r*), and finally calculated the neuropil subtracted traces as the output traces (*F*_*M*_-*rF*_*N*_).

### Experiments Involving Spatial Neuron Segmentation

We also applied TUnCaT to neurons automatically segmented by shallow U-Net neuron segmentation (SUNS) developed in our previous work (Bao et al., 2021). We compared this two-step spatial segmentation then temporal unmixing approach with CaImAn (Giovannucci et al., 2019), a state-of-the-art one-step spatiotemporal unmixing approach.

#### Shallow U-Net Neuron Segmentation

We used the available Python code at https://github.com/YijunBao/Shallow-UNet-Neuron-Segmentation_SUNS (Bao, 2021) (version 1.1.1) to implement the algorithm of SUNS. We used the trained neural networks and optimized post-processing parameters from the cross-validation of ABO dataset performed in our previous work (Bao et al., 2021).

#### CaImAn

We used the available Python code at https://github.com/flatironinstitute/CaImAn (version 1.6.4) to implement the algorithm of CaImAn. We used the batch version. We used the trained neural networks and optimized parameters from the cross-validation of ABO dataset performed in our previous work (Bao et al., 2021).

#### Evaluation of Unmixing Accuracy on Either All Neurons or on Only Common Neurons

We evaluated the accuracy of unmixing in two approaches. In the first approach, we evaluated the accuracy of the detected transients on both true positive neurons and false neurons for each algorithm. In the second approach, we evaluated the accuracy of the detected transients on only neurons commonly found by the GT masks, SUNS, and CaImAn.

We matched two sets of neurons by their Intersection-over-Union (IoU) metrics (Soltanian-Zadeh et al., 2019). We defined the IoU between two masks, m_1_ and m_2_, as:


$$\text{IoU}\,\left({\text{m}}_{1},{\text{m}}_{2}\right)=\frac{\left|{\text{m}}_{1}\cap {\text{m}}_{2}\right|}{\left|{\text{m}}_{1}\cup {\text{m}}_{2}\right|}.$$

We calculated the distance Dist between any pair of masks from set A (${\text{m}}_{i}^{\text{A}}$) and set B (${\text{m}}_{j}^{\text{B}}$) as


$$\mathrm{Dist}\,\left(m_{i}^{A},m_{j}^{B}\right)$$

$$=\left\{ \begin{array}{cc} 1-\mathrm{IoU}\,\left(m_{i}^{A},m_{j}^{B}\right), & \mathrm{IoU}\,\left(m_{i}^{A},m_{j}^{B}\right)\geq 0.5\\ 2, & \mathrm{IoU}\,\left(m_{i}^{A},m_{j}^{B}\right)<0.5 \end{array}\right.$$

In the above equation, Dist = 2 denoted masks that could not be matched due to their small IoU score. Next, we applied the Hungarian algorithm to solve the linear assignment problem using the above distance matrix and defined the paired masks whose distances were smaller than 2 as matched masks.

We used the above matching process to match sets of spatially segmented neurons to the manually curated GT neurons. Algorithm-generated neurons matched to any GT neuron were true positive neurons; algorithm-generated neurons not matched to any GT neurons were false positive neurons, and the GT neurons not matched were false negative neurons. When evaluating the unmixing accuracy, we considered all transients from GT and algorithm-generated neurons. Transients from true positive neurons were classified in the same way as in section “Evaluation Metrics.” Transients from false positive neurons were assessed as false positive transients, contributing to lower precision. Transients from false negative neurons were assessed as false negative transients, contributing to lower recall.

We performed a separate evaluation of unmixing accuracy that de-emphasized the spatial segmentation of neurons. We performed this evaluation by considering transients from only neurons commonly found by the GT masks, SUNS, and CaImAn. These common neurons from the three sets spatially matched to each other under the IoU criterion above. We found the matched neurons by searching the set of neurons matched between GT masks and SUNS masks and the set of neurons matched between GT masks and CaImAn masks. We identified GT neurons matched to both a SUNS mask and a CaImAn mask. If these SUNS and CaImAn masks matched to each other, then they formed a common neuron with the GT mask. We evaluated the unmixing accuracy by only considering the transients in these common neurons and ignoring all transients from other neurons.

### Speed Analysis

The processing time measurement started after all the data were loaded into memory, so the hard drive reading and writing time was excluded. The processing time for SNR videos also included the time used to generate SNR videos.

For experiments using two-step spatial neuron segmentation and then temporal unmixing, we summed the processing time of SUNS and the processing time of the temporal unmixing method as the total processing time. The hard drive reading and writing time was still excluded. When comparing the unmixing results of SNR videos, we added the time to generate SNR videos for CaImAn, but did not add that time when SUNS was used, because the SNR conversion was already done in the pre-processing of SUNS.

## Results

### Temporal Unmixing of Calcium Traces Based on Targeted Non-negative Matrix Factorization

The traces of neurons calculated by averaging all pixels within the neuron masks often contain activity from multiple calcium sources, including the neuron of interest, neighboring neurons, axons, dendrites, and background fluctuations (Figures 1A–C). In the temporal domain, we modeled the measured traces as linear superpositions of the underlying fluorescence intensities from each neural structure and the background; this superposition was represented as mixing by matrix multiplication (Figure 1C, bottom). TUnCaT solves the inverse problem, or unmixing, which estimates the underlying uncontaminated fluorescence intensities from the measured traces.

Temporal unmixing of calcium traces had four stages: auxiliary mask generation, trace calculation, background subtraction, and NMF unmixing (Figure 1D and Supplementary Figure 1, section “Materials and Methods”). First, we defined a background mask surrounding each neuron of interest and an outside mask region that included all pixels within the background region but not belonging to any neuron mask (Figure 1B). This outside region included all calcium sources other than the labeled neurons, such as axons, dendrites, and occasionally unlabeled neurons. Second, we calculated the raw traces of all neurons and their corresponding background and outside regions. Third, we applied background subtraction to all raw traces to eliminate background fluctuations. Fourth, we decontaminated the false transients arising from neighboring neurons, axons, and dendrites by using NMF unmixing. Because each NMF input trace originated from a mask with physical meaning, we required that none of the NMF output traces be identically zero due to over-regularization. We adopted a regularization strategy with a floating NMF regularization parameter, α, to avoid this outcome (section “Materials and Methods”). Finally, we selected the output trace of the neuron of interest by matching each unmixed output trace to its most similar input trace (Supplementary Table 1, section “Materials and Methods”). We parallelized the calculations in each stage over multiple neurons of interest to improve the speed.

### Temporal Unmixing of Calcium Traces Can Unmix Neuron Traces From Experimental Two-Photon Videos Accurately and Quickly

We first evaluated the performance of TUnCaT through leave-one-out cross-validation on the same set of movies and manual labels of neurons from the ABO dataset used in our previous work (section “Materials and Methods,” Supplementary Figures 2A,B; Soltanian-Zadeh et al., 2019; Bao et al., 2021). We analyzed transients from the neurons in the center 200 × 200 pixel regions of the videos, and manually identified the true transients from these neurons (section “Materials and Methods”). We evaluated our algorithm’s unmixing accuracy by comparing the algorithm-detected transients with the manually identified GT transients. We unmixed the neuron traces extracted from either the raw videos or the SNR videos (section “Materials and Methods”).

**FIGURE 2:**
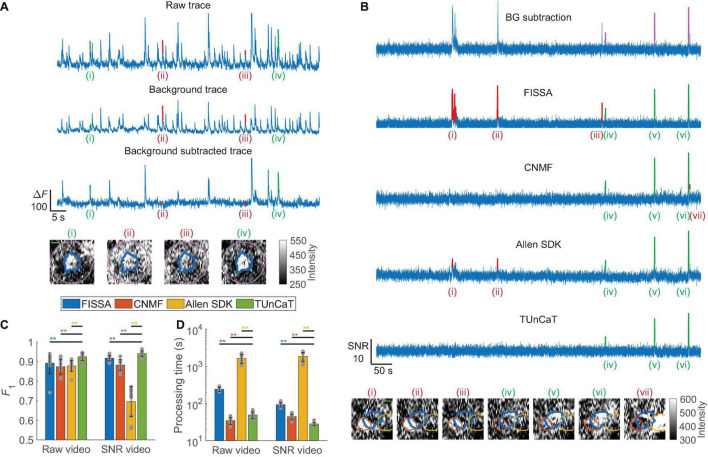
Temporal unmixing of calcium traces (TUnCaT) was more accurate than peer algorithms on experimental two-photon dataset. **(A)** TUnCaT can remove false transients from the raw trace (top) caused by background fluctuation (middle). The background-subtracted trace (bottom) correctly retained two true neuron transients [(i) and (iv), green] of four example transients detected in the raw trace, and suppressed two transients caused by background fluctuations [(ii) and (iii), red]. Each image below the traces is the corresponding spatial profile of fluorescence at the peak of each transient. The thick blue contour in each image shows the boundary of the neuron, and the purple contour shows the background region surrounding the segmented neuron (scale bar: 5 μm). **(B)** TUnCaT can remove false transients caused by neighboring neurons and dendrites for an example neuron. The first trace is the background-subtracted trace of an example neuron representing the trace before unmixing; the magenta transients show the manually determined GT transients. The four remaining traces are the unmixing results of four different methods (FISSA, CNMF, the Allen SDK, and TUnCaT) from that neuron. Subpanels (i)–(vii) show example transients detected by at least one unmixing algorithm. The neuron transients correctly identified by each algorithm are highlighted green, while transients incorrectly identified by each algorithm as false positives are highlighted red. Transients (i)–(iii) came from a neighboring neuron, and transient (vii) came from a dendrite. Each image below the traces is the corresponding spatial profile of fluorescence at the peak of each transient. The thick blue contour in each image shows the boundary of the neuron, and the orange and yellow contours show the boundary of two different neighboring neurons (scale bar: 5 μm). **(C)** The *F*_1_ scores of TUnCaT during 10-round cross-validation were superior to that of the other methods both when processing raw videos and when processing SNR videos (**p < 0.005, two-sided Wilcoxon signed-rank test, *n* = 10 videos; error bars are standard deviations). The gray dots represent scores for the test data on each round of cross-validation. **(D)** The processing time of TUnCaT was comparable to or faster than the other methods both when processing raw videos and when processing SNR videos (**p < 0.005, two-sided Wilcoxon signed-rank test, *n* = 10 videos; error bars are standard deviations). The gray dots represent the processing times of the test data on each round of cross-validation.

Our background subtraction algorithm was effective at removing false transients arising from background. The raw trace and the background trace from an example neuron had many similar transient features, suggesting strong crosstalk (Figure 2A). The background-subtracted trace removed many of these common features that were false transients caused by background fluctuation. Among the four example transients detected in the raw trace, the background-subtracted trace correctly retained two true neuron transients [(i) and (iv), green] and suppressed two transients caused by background fluctuations [(ii) and (iii), red].

Next, we quantified the performance of our unmixing algorithm against peer unmixing algorithms [FISSA (Keemink et al., 2018), CNMF (Pnevmatikakis et al., 2016; Giovannucci et al., 2019), and the Allen SDK (de Vries et al., 2020), section “Materials and Methods”]. We used CNMF as a temporal unmixing algorithm by seeding CNMF with the same set of neuron masks used for other algorithms. We found that TUnCaT was both more accurate and faster than peer algorithms (Figures 2B–D). Analysis of the example neuron qualitatively showed that TUnCaT removed the false transients caused by either a neighboring neuron or a dendrite, and retained the true transients (Figure 2B). On the other hand, FISSA did not remove the false transients (i)–(iii) caused by a neighboring neuron, CNMF did not remove the false transient (vii) caused by a dendrite, and the Allen SDK did not remove the false transients (i)–(ii) caused by a neighboring neuron. While this was a representative qualitative example, the performance can vary across all traces. To show the overall unmixing accuracy, we quantified the transient detection accuracy in terms of the *F*_1_ score for each algorithm (section “Materials and Methods”). The *F*_1_ of TUnCaT was 0.92 ± 0.02 for raw videos and 0.94 ± 0.01 for SNR videos (mean ± SD, *n* = 10 videos), significantly higher than those of other algorithms (*p* = 0.002, two-sided Wilcoxon signed-rank test, *n* = 10 videos; Figure 2C and Supplementary Tables 2A,B). The *F*_1_ scores of TUnCaT were nearly invariant when repeating the analysis multiple times.

The performance of NMF unmixing depended on its regularization parameter, α. Increasing α from small values progressively suppressed the amplitude of the false transients. However, large α values led to over-regularization and consequently inaccurate waveforms in the unmixed traces, such as merged activity from multiple neurons, a flat baseline, or even an output of identically zero (Supplementary Figure 3A). Intermediate α values optimized the unmixing accuracy (Supplementary Figures 3B,C). Our floating α strategy prevented identically zero output traces compared to the fixed α strategy if the manually set α values were too high (*p* < 0.01 for α ≥ 100, two-sided Wilcoxon signed-rank test, *n* = 10 videos; Supplementary Figures 3B,C). However, the final α’s of all neurons were the same as the initial α’s when the initial α’s were optimized values obtained through cross-validation.

In addition to having high detection accuracy, TUnCaT also had speed that was faster than or nearly equal to that of peer algorithms. On a single desktop, TUnCaT processed videos with 56 ± 7 neurons over ∼23,000 frames in 49 ± 10 s for raw videos or 28 ± 3 s for SNR videos (mean ± SD over *n* = 10 videos), significantly faster than the other algorithms when processing SNR videos, and significantly faster than FISSA and the Allen SDK when processing raw videos, although significantly slower than CNMF when processing raw videos (*p* < 0.004, two-sided Wilcoxon signed-rank test; *n* = 10 videos; Figure 2D and Supplementary Tables 2C,D). TUnCaT was also superior in speed when processing videos with dimensions typical of two-photon recordings (Supplementary Figure 4; section “Materials and Methods”); this speed was much faster than the video recording rate.

The processing time of NMF unmixing depended on α. Increasing α reduced the number of iterations required to reach NMF convergence, and thus reduced the processing time (Supplementary Figures 3D,E). However, our floating α strategy tested additional α values if the initial regularization parameter was large. Therefore, the processing time of the floating α strategy was longer than the processing time of strategies using a fixed α if the initial α caused identically zero output traces. Temporal downsampling improved the unmixing speed without sacrificing accuracy at moderate downsampling ratios (Supplementary Figures 5A,B; section “Materials and Methods”).

**FIGURE 3:**
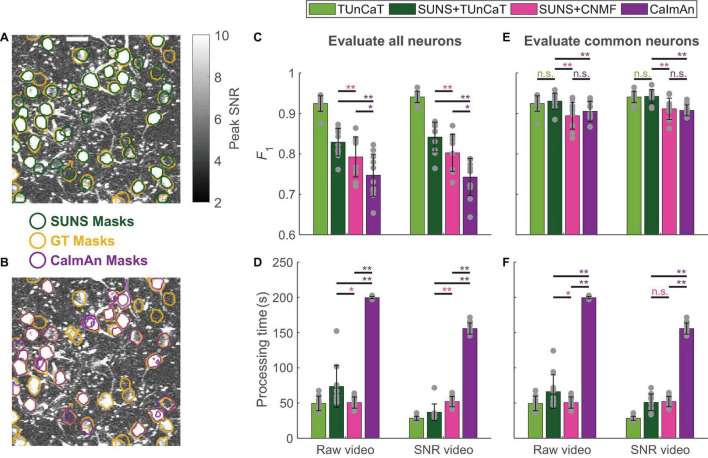
Two-step spatial segmentation and temporal unmixing was more accurate than one-step spatiotemporal unmixing. We also showed the results of applying TUnCaT on GT neurons as a reference, whose processing time did not include manual neuron segmentation. **(A,B)** The SUNS masks matched the GT masks better than the CaImAn masks. The neurons in an example ABO video found by manual labeling as GT (yellow) and **(A)** SUNS (dark green) or **(B)** CaImAn (purple) are overlaid on top of the imaging data. The grayscale images are the projection of the maximum pixel-wise SNR (Scale bar: 20 μm). **(C)** The *F*_1_ scores of CaImAn during 10-round cross-validation were significantly lower than that of SUNS + TUnCaT and SUNS + CNMF both when processing raw videos and when processing SNR videos (*p < 0.05, **p < 0.005, two-sided Wilcoxon signed-rank test, *n* = 10 videos; error bars are standard deviations). We evaluated the accuracy by considering transients from all ground truth and algorithm-generated neurons. The gray dots represent scores for the test data on each round of cross-validation. **(D)** The processing time of CaImAn was significantly longer than SUNS + TUnCaT and SUNS + CNMF both when processing raw videos and when processing SNR videos (*p < 0.05, **p < 0.005, two-sided Wilcoxon signed-rank test, *n* = 10 videos; error bars are standard deviations). We used the α of TUnCaT that optimized the *F*_1_ scores considering transients from all ground truth and algorithm-generated neurons. The gray dots represent the processing times for the test data on each round of cross-validation. **(E)** The *F*_1_ scores of SUNS + TUnCaT during 10-round cross-validation were significantly higher than that of CaImAn and SUNS + CNMF both when processing raw videos and when processing SNR videos (**p < 0.005, n.s. - not significant, two-sided Wilcoxon signed-rank test, *n* = 10 videos; error bars are standard deviations). We evaluated the accuracy by considering only neurons spatially segmented by all methods. The gray dots represent scores for the test data on each round of cross-validation. **(F)** The processing time of CaImAn was significantly longer than SUNS + TUnCaT and SUNS + CNMF both when processing raw videos and when processing SNR videos (*p < 0.05, **p < 0.005, n.s. - not significant, two-sided Wilcoxon signed-rank test, *n* = 10 videos; error bars are standard deviations). We used the α of TUnCaT that optimized the *F*_1_ scores considering only neurons spatially segmented by all methods. The gray dots represent the processing times for the test data on each round of cross-validation.

Practical neuroscience experiments require both spatial segmentation and temporal unmixing. Performing these two tasks with simultaneous spatial and temporal unmixing is convenient. However, a two-step combination of SUNS from our previous work (Bao et al., 2021), for spatial neuron segmentation, and TUnCaT, for temporal unmixing of activity from spatial segmentations, offered superior accuracy and speed. We compared the performance of SUNS + TUnCaT with CaImAn (Giovannucci et al., 2019), a current state-of-the-art spatiotemporal unmixing algorithm, using the same ABO dataset (Figure 3; section “Materials and Methods”). We also evaluated SUNS + CNMF as an intermediate between the previous two approaches, because CNMF and CaImAn shared the same temporal unmixing algorithm. Because SUNS had superior spatial segmentation accuracy compared to that of CaImAn (Figures 3A,B; Bao et al., 2021), we suspected that SUNS would provide a better foundation for obtaining accurate temporal activity traces.

Overall, the two-step processes using SUNS for spatial segmentation and another algorithm for temporal unmixing performed better than the one-step CaImAn algorithm in speed and accuracy. When considering calcium transients from all GT and algorithm-generated neurons, the *F*_1_ scores of SUNS + TUnCaT were significantly higher than the *F*_1_ scores of SUNS + CNMF (*p* = 0.002, two-sided Wilcoxon signed-rank test, *n* = 10 videos; Figure 3C; section “Materials and Methods”), matching our previous comparisons between TUnCaT and seeded CNMF. In addition, the *F*_1_ scores of SUNS + CNMF were significantly higher than the *F*_1_ scores of CaImAn (*p* = 0.02, two-sided Wilcoxon signed-rank test, *n* = 10 videos; Figure 3C). This result occurred because SUNS’s spatial segmentations were more accurate than CaImAn’s spatial segmentations. We also de-emphasized the accuracy of the spatial segmentations by calculating the *F*_1_ scores only on neurons commonly found by manual labeling, SUNS, and CaImAn. Among these neurons, SUNS + TUnCaT still had superior *F*_1_ scores compared to the *F*_1_ scores of SUNS + CNMF and CaImAn (*p* = 0.002, two-sided Wilcoxon signed-rank test, *n* = 10 videos; Figure 3E); the latter two options had similar accuracy (*p* > 0.08, two-sided Wilcoxon signed-rank test, *n* = 10 videos; Figure 3E). Furthermore, SUNS + TUnCaT and SUNS + CNMF were significantly faster than CaImAn (*p* = 0.002, two-sided Wilcoxon signed-rank test, *n* = 10 videos; Figures 3D,F), drawing on SUNS’s higher spatial segmentation speed compared to CaImAn’s speed.

### Temporal Unmixing of Calcium Traces Can Unmix Neuron Traces From Simulated Two-Photon Videos Accurately and Quickly

To evaluate the accuracy of unmixing algorithms with a simulated GT, we simulated two-photon videos using NAOMi (Song et al., 2021) with typical parameters used during video-rate two-photon imaging (section “Materials and Methods”; Supplementary Figure 2C). We identified GT transients from the GT traces exported by NAOMi, and evaluated our algorithm’s unmixing accuracy by comparing the algorithm-detected transients with the GT transients (section “Materials and Methods”).

**FIGURE 4:**
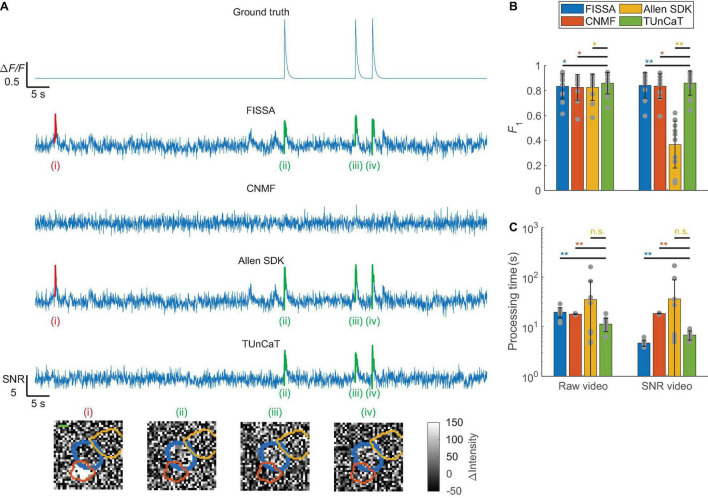
Temporal unmixing of calcium traces (TUnCaT) was more accurate than peer algorithms when processing simulated two-photon videos. **(A)** TUnCaT removed false transients caused by neighboring neurons. The first trace is the ground truth trace exported from the simulation process. The four remaining traces are the unmixed traces of the neuron of interest from four different methods (FISSA, CNMF, the Allen SDK, and TUnCaT). The labels (i)–(iv) indicate examples transients correctly (green) or incorrectly (red) detected by at least one unmixing algorithm. Each image below the traces is the corresponding spatial profile of fluorescence at the peak of each transient. We subtracted the median image over time from each image to remove the static fluorescence. The thick blue contour in each image shows the boundary of the neuron of interest, and the orange and yellow contours show the boundary of two different neighboring neurons (scale bar: 5 μm). **(B)** The *F*_1_ scores of TUnCaT during 10-round cross-validation were superior to that of the other methods both when processing raw videos and when processing SNR videos (*p < 0.05, **p < 0.005, two-sided Wilcoxon signed-rank test, *n* = 10 videos; error bars are standard deviations). The gray dots represent scores for the test data on each round of cross-validation. **(C)** The processing time of TUnCaT was comparable to or faster than the other methods both when processing raw videos and when processing SNR videos (**p < 0.005, n.s. - not significant, two-sided Wilcoxon signed-rank test, *n* = 10 videos; error bars are standard deviations). The gray dots represent the processing times for the test data on each round of cross-validation.

When processing these simulated data, we found that TUnCaT was both more accurate and faster than peer algorithms (Figure 4). Analysis of the example neuron qualitatively showed that TUnCaT removed the false transients caused by a neighboring neuron and retained the true transients (Figure 4A). On the other hand, FISSA and the Allen SDK did not remove the false transient (i) caused by a neighboring neuron, and CNMF removed the true transients (ii)–(iv). Over all videos, the *F*_1_ of TUnCaT was 0.86 ± 0.09 for raw videos and 0.86 ± 0.10 for SNR videos (mean ± SD, *n* = 10 videos), significantly higher than those of other algorithms (*p* < 0.02, two-sided Wilcoxon signed-rank test, *n* = 10 videos; Figure 4B and Supplementary Tables 3A,B).

In addition to high detection accuracy, the speed of TUnCaT was faster than or nearly equal to the speed of peer algorithms. On a single desktop, TUnCaT processed videos with 35 ± 10 neurons over 3,000 frames in 11 ± 3 s for raw videos or 7 ± 1 s for SNR videos (mean ± SD over *n* = 10 videos), faster than the other algorithms when processing raw videos, and faster than CNMF and the Allen SDK when processing SNR videos, although slower than FISSA when processing SNR videos (*p* > 0.23 compared to the Allen SDK, and *p* < 0.004 compared to FISSA and CNMF, two-sided Wilcoxon signed-rank test, *n* = 10 videos; Figure 4C and Supplementary Tables 3C,D). Our temporal downsampling strategy again significantly improved the processing speed without sacrificing accuracy at moderate downsampling ratios for these simulated videos (Supplementary Figures 5C,D).

To test whether the success of TUnCaT depended on the simulation parameters, we simulated additional conditions over an expanded parameter space of two-photon imaging (Supplementary Figure 6): some portions of this parameter space, such as lower frame rates, longer video lengths, lower laser power, and different sensor types, have been widely used in the neuroscience community; other portions of this parameter space, such as higher frame rates, shorter videos, larger densities of neurons, and higher noise, are accessible by custom experiments, but are not typically encountered in experimental settings. The superiority of TUnCaT was generalizable to a variety of conditions: the *F*_1_ of TUnCaT was either the highest or close to the highest; the processing speed of TUnCaT was generally faster than or comparable to the speed of other algorithms.

Up to now, we used cross-validation and the GT transients to optimize the regularization parameter α. Fortunately, the optimal α in all our test datasets were all close to 1 (Supplementary Table 4). If we set α = 1 and used the floating α strategy, the resulting *F*_1_ scores under a wide range of simulated imaging conditions were generally very close to the *F*_1_ scores when using α optimized through cross-validation for each imaging condition (Supplementary Figure 7). Thus, α = 1 could be a good starting point that allows users to potentially bypass cross-validation if manual labels are not available. However, cross-validation would still be more robust against the idiosyncrasies of new imaging conditions, such as videos with very large noise scale.

### Temporal Unmixing of Calcium Traces Can Unmix Neuron Traces From Experimental One-Photon Videos Accurately

**FIGURE 5:**
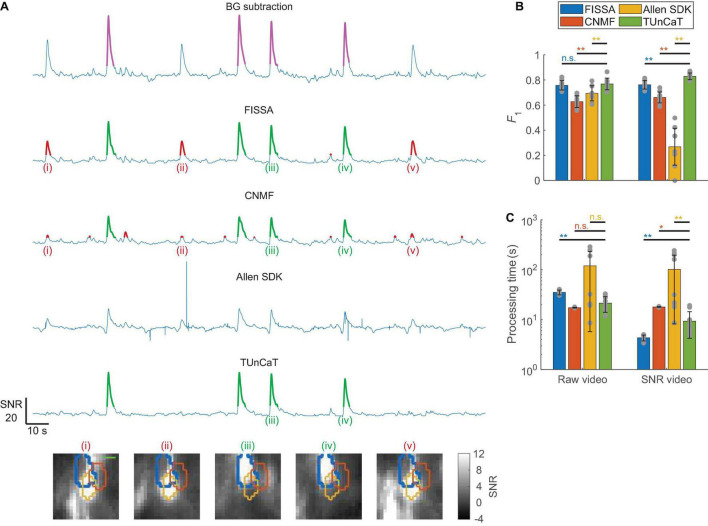
Temporal unmixing of calcium traces (TUnCaT) was more accurate than peer algorithms when processing experimental one-photon videos. **(A)** TUnCaT can remove false transients caused by neighboring neurons. The first trace is the background-subtracted trace of the SNR video of an example neuron representing the trace before unmixing, and the magenta transients show the manually determined ground truth transients. The four remaining traces are the unmixed traces of that neuron from four different methods (FISSA, CNMF, the Allen SDK, and TUnCaT). The labels (i)–(v) indicate example transients correctly (green) or incorrectly (red) detected by at least one unmixing algorithm. Each image below the traces is the corresponding SNR image at the peak of each transient. The thick blue contour in each image shows the boundary of the neuron of interest, and the orange and yellow contours show the boundary of two different neighboring neurons (scale bar: 10 μm). **(B)** The *F*_1_ scores of TUnCaT during 9-round cross-validation were superior to that of the other methods both when processing raw videos and when processing SNR videos (**p < 0.005, n.s. - not significant, two-sided Wilcoxon signed-rank test, *n* = 9 videos; error bars are standard deviations). The gray dots represent scores for the test data on each round of cross-validation. **(C)** The processing time of TUnCaT was comparable to or faster than the other methods both when processing raw videos and when processing SNR videos (*p < 0.05, **p < 0.005, n.s. - not significant, two-sided Wilcoxon signed-rank test, *n* = 9 videos; error bars are standard deviations). The gray dots represent the processing times for the test data on each round of cross-validation.

We further tested whether TUnCaT extract clean temporal traces from experimental one-photon videos (section “Materials and Methods,” Supplementary Figure 2D). We manually identified GT transients from the GT neurons, and evaluated our algorithm’s unmixing accuracy by comparing the algorithm-detected transients with the GT transients (section “Materials and Methods”). We found that TUnCaT was more accurate than peer algorithms (Figures 5A,B). Analysis of an example neuron qualitatively showed that TUnCaT removed the false transients caused by a neighboring neuron and retained the true transients (Figure 5A). On the other hand, FISSA and CNMF did not remove the false transients (i), (ii), and (v) caused by a neighboring neuron. The unmixed trace from the Allen SDK had some extremely large absolute values at some time points (saturated in the figure), and our transient detection algorithm failed to detect any transient. Over all videos, the *F*_1_ of TUnCaT was 0.77 ± 0.05 for raw videos and 0.83 ± 0.03 for SNR videos (mean ± SD, *n* = 9 videos), generally significantly higher than other algorithms (*p* = 0.25 compared to FISSA when processing raw videos, and *p* = 0.004 for other comparisons, two-sided Wilcoxon signed-rank test, *n* = 9 videos; Figure 5B and Supplementary Tables 5A,B).

In addition to high detection accuracy, the speed of TUnCaT was faster than or comparable to peer algorithms. On a single desktop, TUnCaT processed videos with 38 ± 8 neurons over 6,000 frames in 21 ± 7 s for raw videos or 9 ± 5 s for SNR videos (mean ± SD over *n* = 9 videos), significantly faster than FISSA when processing raw videos, significantly faster than CNMF and the Allen SDK when processing SNR videos, not significantly different from CNMF and the Allen SDK when processing raw videos, although significantly slower than FISSA when processing SNR videos (*p* = 0.004 compared to FISSA, *p* < 0.02 compared to the CNMF and Allen SDK when processing SNR videos, and *p* > 0.09 compared to the CNMF and Allen SDK when processing raw videos, two-sided Wilcoxon signed-rank test, *n* = 9 videos; Figure 5C and Supplementary Tables 5C,D). The relationships between FISSA’s regularization parameter α and FISSA’s accuracy or speed were similar to the equivalent relationships for TUnCaT: large α values shortened processing times and generally removed false transients, but also risked generating inaccurate calcium waveforms through over-regularization. The optimal α of FISSA when processing SNR videos likely entered this over-regularization regime. As a result, when processing SNR videos, FISSA led TUnCaT in speed, but lagged in accuracy. Similar to previous datasets, our temporal downsampling strategy again significantly improved the processing speed without sacrificing accuracy at moderate downsampling ratios (Supplementary Figures 5E,F).

## Discussion

We present in this paper TUnCaT, an automated, fast, and accurate algorithm to calculate the temporal calcium signals of individual neurons from fluorescence imaging movies. TUnCaT accurately removed false transients caused by neighboring neurons, axons, dendrites, and large-scale background fluctuations. TUnCaT had four stages: auxiliary mask generation, trace calculation, background subtraction, and NMF unmixing. We compared the accuracy and speed of TUnCaT with peer algorithms on multiple datasets with various imaging techniques and conditions, including experimental two-photon videos, simulated two-photon videos with varying simulation parameters, and experimental one-photon videos. TUnCaT consistently achieved the highest accuracy represented by high *F*_1_ scores, and its speed was faster than or comparable to other algorithms.

Temporal unmixing of calcium traces had some similarities with FISSA (Keemink et al., 2018); both algorithms first calculated the traces of the neuron of interest and potentially contaminating regions, and then used NMF to unmix the traces in the temporal domain. TUnCaT also differed from FISSA in several key ways: (1) TUnCaT used an explicit background subtraction step to remove background fluctuations before NMF unmixing; (2) The contaminating traces used in TUnCaT targeted neighboring neuron regions and an outside region containing axons and dendrites; each region had a clear physical basis, and the regions could overlap; (3) TUnCaT employed a floating α strategy to avoid identically zero output traces, a sign of over-regularization; (4) TUnCaT used parallel computing in trace calculation, which significantly improved the speed when processing imaging videos with large numbers of pixels and frames (e.g., ABO videos); (5) Optional temporal downsampling significantly improved the speed of TUnCaT without sacrificing accuracy. These TUnCaT features enabled high accuracy and short processing time.

Temporal unmixing of calcium traces had two conceptual differences compared to three-dimensional spatiotemporal unmixing algorithms. First, TUnCaT can use spatial neuron masks from any approach, including manual labels or masks generated by any automatic neuron segmentation algorithm. Recently developed neuron segmentation algorithms based on deep learning have outperformed spatiotemporal unmixing algorithms in the accuracy of the neuron spatial footprints, and were fast (Soltanian-Zadeh et al., 2019; Bao et al., 2021). Users could integrate such accuracy and speed with the accuracy and speed of TUnCaT. Second, TUnCaT had good generalized performance when processing imaging data using different calcium sensor types and imaging conditions, in raw or SNR formats. This performance likely resulted from TUnCaT making minimal assumptions about the calcium fluorescence waveforms: the algorithm presumed that signals were non-negative and mixed linearly. Existing spatiotemporal methods added additional assumptions such as the transient shape and decay dynamics, which vary greatly and non-linearly with respect to the underlying neural activity in experimental settings.

In addition to raw videos, we also tested TUnCaT and its peer unmixing algorithms on SNR videos (section “Materials and Methods”; Bao et al., 2021). Our previous work demonstrated that segmentations of active neurons from SNR videos were more accurate than segmentations from raw videos (Bao et al., 2021). SNR videos showed the transients of neurons more clearly, because they removed spatially large background fluctuations, enhanced the SNR of calcium transient waveforms, and de-emphasized static periods. Our results identifying active transients from temporal traces in this paper matched the spatial segmentation results in our previous work: all unmixing methods except the Allen SDK had higher *F*_1_ scores when processing SNR videos than when processing raw videos; FISSA and TUnCaT also had shorter processing times when using SNR videos. In particular, one-photon imaging data, having high static fluorescence, required the SNR representation to clearly distinguish active neurons.

Temporal unmixing of calcium traces worked for both two-photon and one-photon videos. In general, two-photon microscopy had high optical sectioning that sharply defined the spatial footprints of individual neurons, minimized neuron overlap, and minimized background. Two-photon videos thus clearly defined the spatial footprints of neurons. This higher spatial resolution translated to a more tractable unmixing problem, as all algorithms had superior accuracy metrics when processing two-photon videos than when processing one-photon videos. On the contrary, one-photon microscopy suffers from tissue scattering and low optical sectioning. Neural structures imaged in one-photon microscopy appear larger than their true spatial footprints through blurring and are contaminated by out-of-focus emission. These mixing processes caused neurons in one-photon videos to have significant overlap with neighboring neural structures and suffer from large background fluctuations. TUnCaT’s accurate unmixing and background subtraction partially alleviated these issues. Additional tuning of the regularization parameter α for each individual neuron could further improve the unmixing accuracy.

A natural future extension of TUnCaT is the development of online trace unmixing. Modern neuroscience experiments seek to apply real-time behavioral or neural manipulation in response to the recorded neural activity (Sitaram et al., 2017; Kearney et al., 2019). Such experiments demand online decontamination of recorded calcium activity on a frame-by-frame basis during the video acquisition, with minimal delay between the recording of the data to the output of the neural activity, and without knowledge of future signals. The high batch processing speed of TUnCaT sets the foundation for processing each frame faster than the frame acquisition rate. A combination of our processing techniques with online NMF (Giovannucci et al., 2017; Friedrich et al., 2021) could meet the demands of online neuroscience experiments after initial ramp-up calculations. Our downsampling strategy could generate additional computation headroom by reducing the frequency of calculating the mixing matrix.

Our strategy of targeting unmixing to physically relevant signal sources has similar applications in other unmixing or decomposition problems in the broad neuroscience field, such as mesoscopic calcium imaging, multispectral fluorescence imaging, and electroencephalography (EEG). For example, the unmixing of signals from mesoscopic calcium imaging of the mouse cortical surface benefitted from established structurally defined brain regions that regularized the unmixing process and led to more interpretable and consistent representations (Saxena et al., 2020). In multispectral fluorescence imaging, spectral unmixing benefitted from an approximate global target/background classification determined using graph cut, because NMF initialized with the spatial distribution of the targets helped constrain the sparsity of the targets (Qin et al., 2016). In EEG, source localization with the knowledge of source distribution (Hansen et al., 2019) or brain segmentation (Biscay et al., 2018) helped to unmix the signals and accurately compute neural dynamics. Overall, closer collaboration between the algorithms developed for structural segmentation and the algorithms developed for calculating dynamics could produce mutually beneficial increases in accuracy.

## Data Availability Statement

Code for TUnCaT can be accessed in https://github.com/YijunBao/TUnCaT (doi: 10.5281/zenodo.5764575). All data and code used for running experiments and plotting are available at https://figshare.com/articles/dataset/TUnCaT_paper_reproduction/17132069 (doi: 10.6084/m9.figshare.17132069.v1). We used a publicly available dataset provided by the Allen Institute on https://github.com/AllenInstitute/AllenSDK/wiki/Use-the-Allen-Brain-Observatory—Visual-Coding-on-AWS, and we used the corresponding manual labels created from our previous work, https://github.com/soltanianzadeh/STNeuroNet/tree/master/Markings/ABO.

## Ethics Statement

The animal study was reviewed and approved by Duke Institutional Animal Care and Use Committee.

## Author Contributions

YG conceived and designed the project. YB and YG developed the algorithm of TUnCaT, analyzed the data, and wrote the manuscript. YB implemented the code of TUnCaT and ran the unmixing experiment. AA and YB implemented the graphical user interface to manually label transients. ER recorded the experimental one-photon videos and labeled the corresponding neuron masks. All authors reviewed the manuscript and approved this work.

## Conflict of Interest

The authors declare that the research was conducted in the absence of any commercial or financial relationships that could be construed as a potential conflict of interest.

## Publisher’s Note

All claims expressed in this article are solely those of the authors and do not necessarily represent those of their affiliated organizations, or those of the publisher, the editors and the reviewers. Any product that may be evaluated in this article, or claim that may be made by its manufacturer, is not guaranteed or endorsed by the publisher.
