# Supplementary material for: Decontaminate Traces From Fluorescence Calcium Imaging Videos Using Targeted Non-negative Matrix Factorization
Source: Front Neurosci. 2022 Jan 21;15:797421. doi: 10.3389/fnins.2021.797421 (PMC8815790; doi:10.3389/fnins.2021.797421)
Supplement: Supplementary file 1 [file Table_1.pdf]

Supplementary Tables

Supplementary Table 1. A demonstration of the process to match unmixed traces F_{sep} with input traces F_{meas} by analyzing the mixing matrix M. Four quantities were involved in this procedure: input and output matrices M and F_{sep}, as well as two helper matrices M_0 and P. Each row of this table was one operation and showed the quantities updated in this operation.

**Operations**	M	F_{sep}	M_0	P
Inputs	$\begin{pmatrix} 1 & 1 & 9 \\ 3 & 1 & 5 \\ 0 & 2 & 6 \end{pmatrix}$	$\begin{pmatrix} c_1 \\ c_2 \\ c_3 \end{pmatrix}$		
(1) Column-wise normalization of M	$\begin{pmatrix} 1/4 & 1/4 & 9/20 \\ 3/4 & 1/4 & 1/4 \\ 0 & 1/2 & 3/10 \end{pmatrix}$	$\begin{pmatrix} 4c_1 \\ 4c_2 \\ 20c_3 \end{pmatrix}$		
(2) Initialize M_0 and P			$\begin{pmatrix} 1/4 & 1/4 & 9/20 \\ \mathbf{3/4} & 1/4 & 1/4 \\ 0 & 1/2 & 3/10 \end{pmatrix}$	$\begin{pmatrix} 0 & 0 & 0 \\ 0 & 0 & 0 \\ 0 & 0 & 0 \end{pmatrix}$
(3-1) Find the largest element in M_0: $m_{0(2,1)}$			$\begin{pmatrix} 0 & 1/4 & 9/20 \\ 0 & 0 & 0 \\ 0 & 1/2 & 3/10 \end{pmatrix}$	$\begin{pmatrix} 0 & 0 & 0 \\ 1 & 0 & 0 \\ 0 & 0 & 0 \end{pmatrix}$
Column-wise normalization of M_0			$\begin{pmatrix} 0 & 1/3 & 3/5 \\ 0 & 0 & 0 \\ 0 & \mathbf{2/3} & 2/5 \end{pmatrix}$	
(3-2) Find the largest element in M_0: $m_{0(3,2)}$			$\begin{pmatrix} 0 & 0 & 3/5 \\ 0 & 0 & 0 \\ 0 & 0 & 0 \end{pmatrix}$	$\begin{pmatrix} 0 & 0 & 0 \\ 1 & 0 & 0 \\ 0 & 1 & 0 \end{pmatrix}$
Column-wise normalization of M_0			$\begin{pmatrix} 0 & 0 & \mathbf{1} \\ 0 & 0 & 0 \\ 0 & 0 & 0 \end{pmatrix}$	
(3-3) Find the largest element in M_0: $m_{0(1,3)}$			$\begin{pmatrix} 0 & 0 & 0 \\ 0 & 0 & 0 \\ 0 & 0 & 0 \end{pmatrix}$	$\begin{pmatrix} 0 & 0 & 1 \\ 1 & 0 & 0 \\ 0 & 1 & 0 \end{pmatrix}$
(4) $M = MP^T$, $F_{sep} = PF_{sep}$	$\begin{pmatrix} 9/20 & 1/4 & 1/4 \\ 1/4 & 3/4 & 1/4 \\ 3/10 & 0 & 1/2 \end{pmatrix}$	$\begin{pmatrix} 20c_3 \\ 4c_1 \\ 4c_2 \end{pmatrix}$		
(5) Diagonal normalization of M	$\begin{pmatrix} 1 & 1/3 & 1/2 \\ 5/9 & 1 & 1/2 \\ 2/3 & 0 & 1 \end{pmatrix}$	$\begin{pmatrix} 9c_3 \\ 3c_1 \\ 2c_2 \end{pmatrix}$		

Supplementary Table 2. The statistical significance of comparisons between any two unmixing methods on accuracy and speed when processing the ABO two-photon dataset. The *p*-values of the two-sided Wilcoxon signed-rank tests performed in **Figure 2** quantified the statistical significance of the differences between the F_1 scores and processing times of any two unmixing methods when processing the experimental two-photon dataset through 10-fold leave-one-out cross-validation.

(A) Tests on the F_1 score for raw videos; $n = 10$.

	**FISSA**	**CNMF**	**Allen SDK**	**TUnCaT**
**FISSA**	-	0.0645	0.0840	0.0020
**CNMF**	0.0645	-	1.0000	0.0020
**Allen SDK**	0.0840	1.0000	-	0.0020
**TUnCaT**	0.0020	0.0020	0.0020	-

(B) Tests on the F_1 score for SNR videos; $n = 10$.

	**FISSA**	**CNMF**	**Allen SDK**	**TUnCaT**
**FISSA**	-	0.0020	0.0020	0.0020
**CNMF**	0.0020	-	0.0020	0.0020
**Allen SDK**	0.0020	0.0020	-	0.0020
**TUnCaT**	0.0020	0.0020	0.0020	-

(C) Tests on the processing time for raw videos; $n = 10$.

	**FISSA**	**CNMF**	**Allen SDK**	**TUnCaT**
**FISSA**	-	0.0020	0.0020	0.0020
**CNMF**	0.0020	-	0.0020	0.0039
**Allen SDK**	0.0020	0.0020	-	0.0020
**TUnCaT**	0.0020	0.0039	0.0020	-

(D) Tests on the processing time for SNR videos; $n = 10$.

	**FISSA**	**CNMF**	**Allen SDK**	**TUnCaT**
**FISSA**	-	0.0020	0.0020	0.0020
**CNMF**	0.0020	-	0.0020	0.0020
**Allen SDK**	0.0020	0.0020	-	0.0020
**TUnCaT**	0.0020	0.0020	0.0020	-

Supplementary Table 3. The statistical significance of comparisons between any two unmixing methods on accuracy and speed when processing the simulated two-photon dataset. The *p*-values of the two-sided Wilcoxon signed-rank tests performed in **Figure 4** quantified the statistical significance of the differences between the F_1 scores and processing times of any two unmixing methods when processing the simulated two-photon dataset through 10-fold leave-one-out cross-validation.

(A) Tests on the F_1 score for raw videos; $n = 10$.

	FISSA	CNMF	Allen SDK	TUnCaT
**FISSA**	-	0.5566	0.1055	0.0195
**CNMF**	0.5566	-	1.0000	0.0098
**Allen SDK**	0.1055	1.0000	-	0.0059
**TUnCaT**	0.0195	0.0098	0.0059	-

(B) Tests on the F_1 score for SNR videos; $n = 10$.

	FISSA	CNMF	Allen SDK	TUnCaT
**FISSA**	-	0.8457	0.0020	0.0020
**CNMF**	0.8457	-	0.0020	0.0195
**Allen SDK**	0.0020	0.0020	-	0.0020
**TUnCaT**	0.0020	0.0195	0.0020	-

(C) Tests on the processing time for raw videos; $n = 10$.

	FISSA	CNMF	Allen SDK	TUnCaT
**FISSA**	-	0.3223	0.8457	0.0020
**CNMF**	0.3223	-	0.6953	0.0039
**Allen SDK**	0.8457	0.6953	-	0.4316
**TUnCaT**	0.0020	0.0039	0.4316	-

(D) Tests on the processing time for SNR videos; $n = 10$.

	FISSA	CNMF	Allen SDK	TUnCaT
**FISSA**	-	0.0020	0.0020	0.0020
**CNMF**	0.0020	-	1.0000	0.0020
**Allen SDK**	0.0020	1.0000	-	0.2324
**TUnCaT**	0.0020	0.0020	0.2324	-

Supplementary Table 4. Optimized parameter *α* of TUnCaT through leave-one-out cross-validation on different datasets and different video types.

	Optimized *α* (mean ± SD)	
**Dataset**	**Raw videos**	**SNR videos**
Experimental two-photon (**Figure 2**, *n* = 10 videos)	0.47 ± 0.09	1.80 ± 0.40
Simulated two-photon (**Figure 4**, *n* = 10 videos)	0.57 ± 0.81	0.61 ± 0.59
Experimental one-photon (**Figure 5**, *n* = 9 videos)	0.30 ± 0.19	1.45 ± 0.82

Supplementary Table 5. The statistical significance of comparisons between any two unmixing methods on accuracy and speed when processing the one-photon dataset. The *p*-values of the two-sided Wilcoxon signed-rank tests performed in **Figure 5** quantified the statistical significance of the differences between the F_1 scores and processing times of any two unmixing methods when processing the experimental one-photon dataset through 9-fold leave-one-out cross-validation.

(A) Tests on the F_1 score for raw videos; n = 9.

	**FISSA**	**CNMF**	**Allen SDK**	**TUnCaT**
**FISSA**	-	0.0039	0.0039	0.2500
**CNMF**	0.0039	-	0.0117	0.0039
**Allen SDK**	0.0039	0.0117	-	0.0039
**TUnCaT**	0.2500	0.0039	0.0039	-

(B) Tests on the F_1 score for SNR videos; n = 9.

	**FISSA**	**CNMF**	**Allen SDK**	**TUnCaT**
**FISSA**	-	0.0039	0.0039	0.0039
**CNMF**	0.0039	-	0.0039	0.0039
**Allen SDK**	0.0039	0.0039	-	0.0039
**TUnCaT**	0.0039	0.0039	0.0039	-

(C) Tests on the processing time for raw videos; n = 9.

	**FISSA**	**CNMF**	**Allen SDK**	**TUnCaT**
**FISSA**	-	0.0039	0.3594	0.0039
**CNMF**	0.0039	-	0.0273	0.2031
**Allen SDK**	0.3594	0.0273	-	0.0977
**TUnCaT**	0.0039	0.2031	0.0977	-

(D) Tests on the processing time for SNR videos; n = 9.

	**FISSA**	**CNMF**	**Allen SDK**	**TUnCaT**
**FISSA**	-	0.0039	0.0039	0.0039
**CNMF**	0.0039	-	0.0273	0.0117
**Allen SDK**	0.0039	0.0273	-	0.0039
**TUnCaT**	0.0039	0.0117	0.0039	-
