# Supplementary material for: Decontaminate Traces From Fluorescence Calcium Imaging Videos Using Targeted Non-negative Matrix Factorization
Source: Front Neurosci. 2022 Jan 21;15:797421. doi: 10.3389/fnins.2021.797421 (PMC8815790; doi:10.3389/fnins.2021.797421)
Supplement: Supplementary file 2 [file Image_1.pdf]

Supplementary Figures

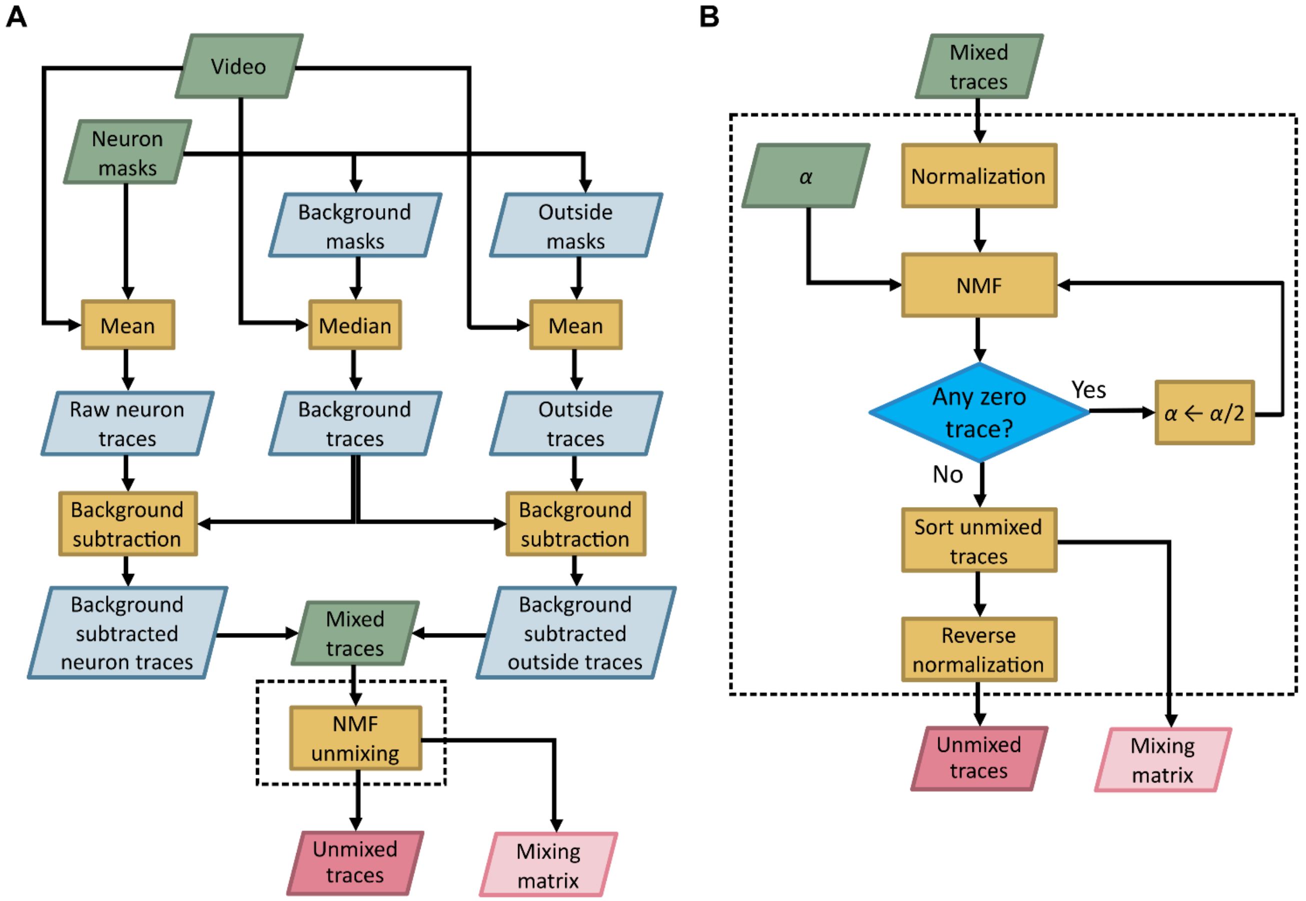

Supplementary Figure 1. Detailed flowchart of TUnCaT.

(A) The overall flowchart of TUnCaT expands on the outline in **Figure 1D**. The inputs are the video and the neuron masks. The outputs are the unmixed traces and the mixing matrix.

(B) The flowchart of NMF unmixing block in **(A)**. The inputs are the mixed traces obtained after background subtraction and a given α. The outputs are the unmixed traces and the mixing matrix.

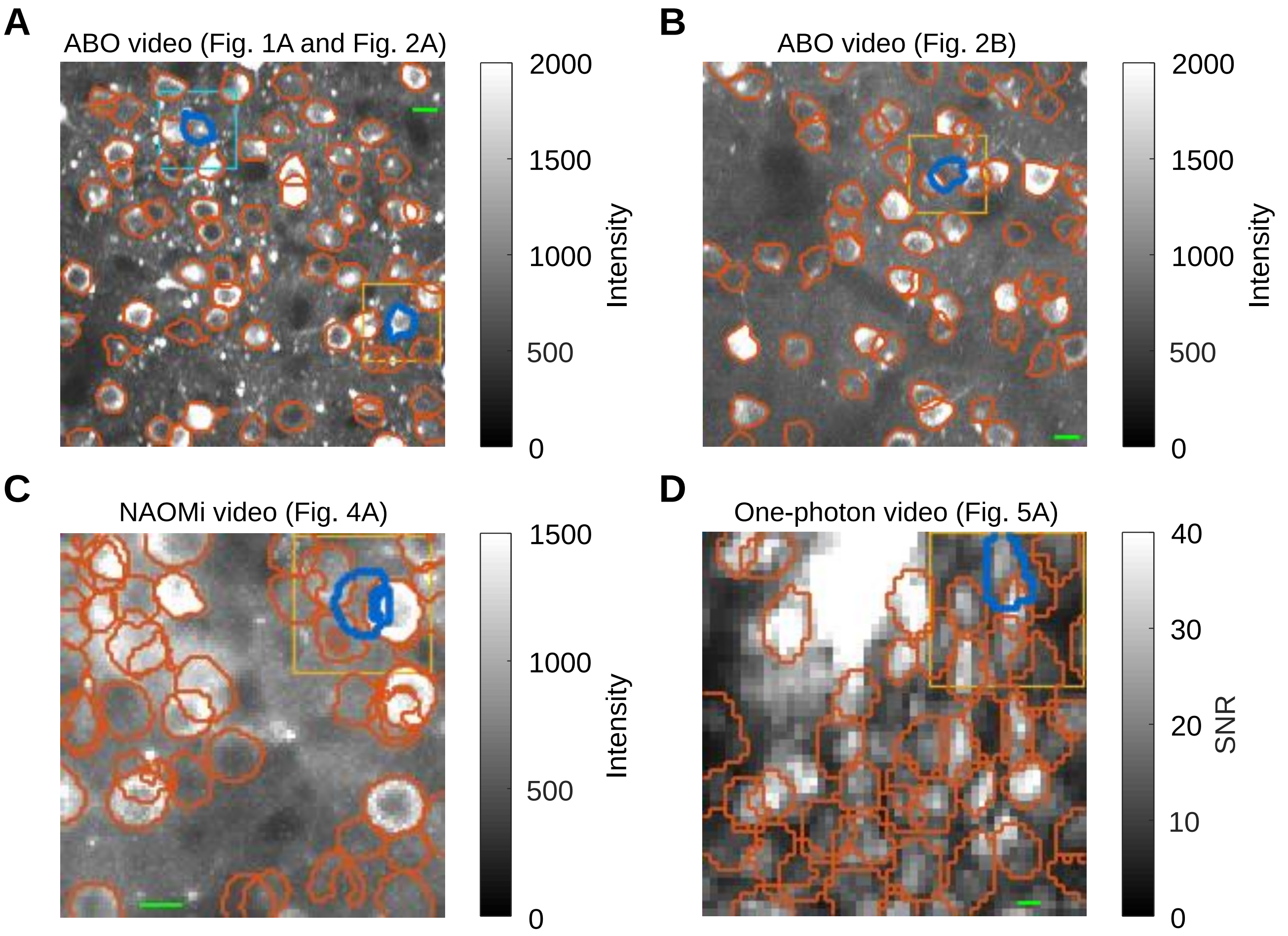

Supplementary Figure 2. Summary images of entire fields of view covering example videos used in the main figures.

(A) The maximum intensity projection image of the video from the ABO dataset used in **Figure 1A** and **Figure 2A**.

(B) The maximum intensity projection image of the video from the ABO dataset used in **Figure 2B**.

(C) The maximum intensity projection image of the video from the simulated NAOMi dataset used in **Figure 4A**.

(D) The maximum intensity projection image of the SNR video from our one-photon dataset used in **Figure 5A**.

In all panels, the yellow and cyan boxed regions contain the neurons shown in the main figures (in **(A)**, the cyan box is for **Figure 1A**, and the yellow box is for **Figure 2A**). The thick blue contour in each image shows the boundary of the neuron of interest. The orange contours in each image show the boundaries of other neurons (scale bars: 10 µm).

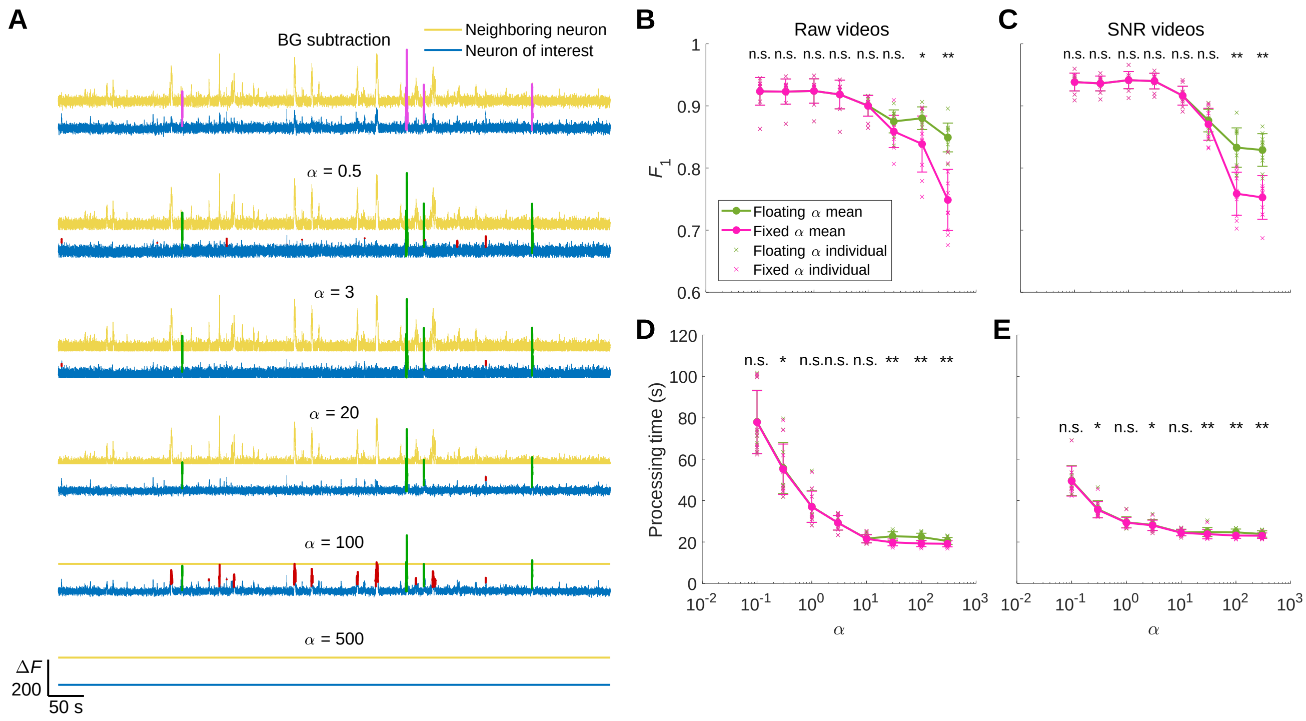

Supplementary Figure 3. The performance of TUnCaT depended on the regularization parameter *α*. A proper regularization parameter *α* optimized the detection of transients within experimental two-photon videos. We compared the unmixing results when using a fixed *α* to our floating *α* strategy initialized with the same *α*. A floating *α* can prevent identically zero output traces at high *α* values and increase the accuracy of detecting transients within experimental two-photon videos.

(A) Each panel shows the traces of the neuron of interest (*blue*) and the neighboring neuron (*yellow*). The first panel shows the background-subtracted traces before unmixing (equivalent to $\alpha = 0$). The magenta transients in the first panel show the manually determined ground truth transients in the trace of the neuron of interest. The five remaining panels are the unmixed traces of TUnCaT using different fixed *α*. Each trace from the neuron of interest highlights the correctly (*green*) or incorrectly (*red*) identified neuron transients. Both the unmixed traces were identically zero when $\alpha = 500$ due to over-regularization.

(B-C) Both when **(B)** processing raw videos and when **(C)** processing SNR videos, the F_1 scores of TUnCaT during 10-round cross-validation increased with *α* at small *α*, but decreased with *α* at large *α*. The F_1 scores when using a floating *α* were significantly higher than the F_1 scores when using a fixed *α* at high *α* values ($\alpha \geq 100$). The labels on top of the curves show the significance of the difference between fixed and floating *α* strategies (*$p < 0.05$, **$p < 0.005$, n.s. - not significant, two-sided Wilcoxon signed-rank test, $n = 10$ videos; error bars are standard deviations).

(D-E) The processing time generally decreased with increasing *α* within experimental two-photon videos both **(D)** when processing raw videos and **(E)** when processing SNR videos. Small increases in processing time occurred at high *α* values when using a floating *α*. The processing times when using a floating *α* were significantly longer than the processing times when using a fixed *α* at high *α* values ($\alpha \geq 30$). The labels on top of the curves show the significance of the difference between fixed and floating

α strategies (*$p < 0.05$, **$p < 0.005$, n.s. - not significant, two-sided Wilcoxon signed-rank test, $n = 10$ videos; error bars are standard deviations).

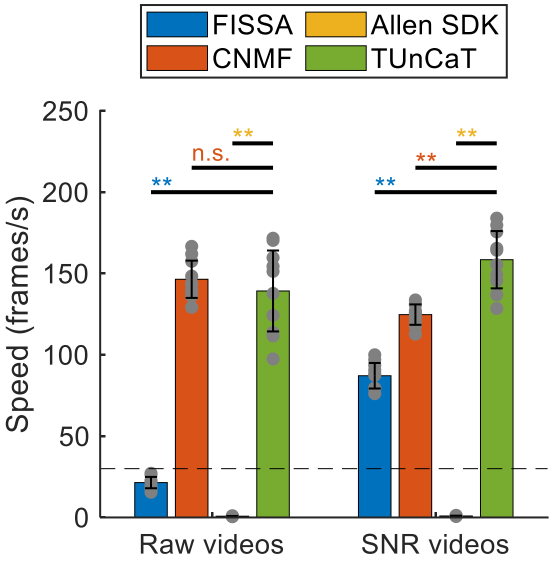

Supplementary Figure 4. TUnCaT was faster than the video recording rate.

The processing speed (frames/s) of TUnCaT on full ABO videos with dimensions typical of two-photon recordings (487 × 487 pixels) was comparable to or faster than FISSA, CNMF, and the Allen SDK both when processing raw videos and when processing SNR videos (**p < 0.005, two-sided Wilcoxon signed-rank test, n = 10 videos; error bars are standard deviations), and was consistently much faster than video recording rate (*black dashed line*). The gray dots represent the processing speed of each video. For FISSA and TUnCaT, we set the α as the mean optimal α over the 10-round cross-validation for the videos cropped to the center 200 × 200 pixels. The speed of the Allen SDK was smaller than 1 frame/s, so its bars are nearly invisible.

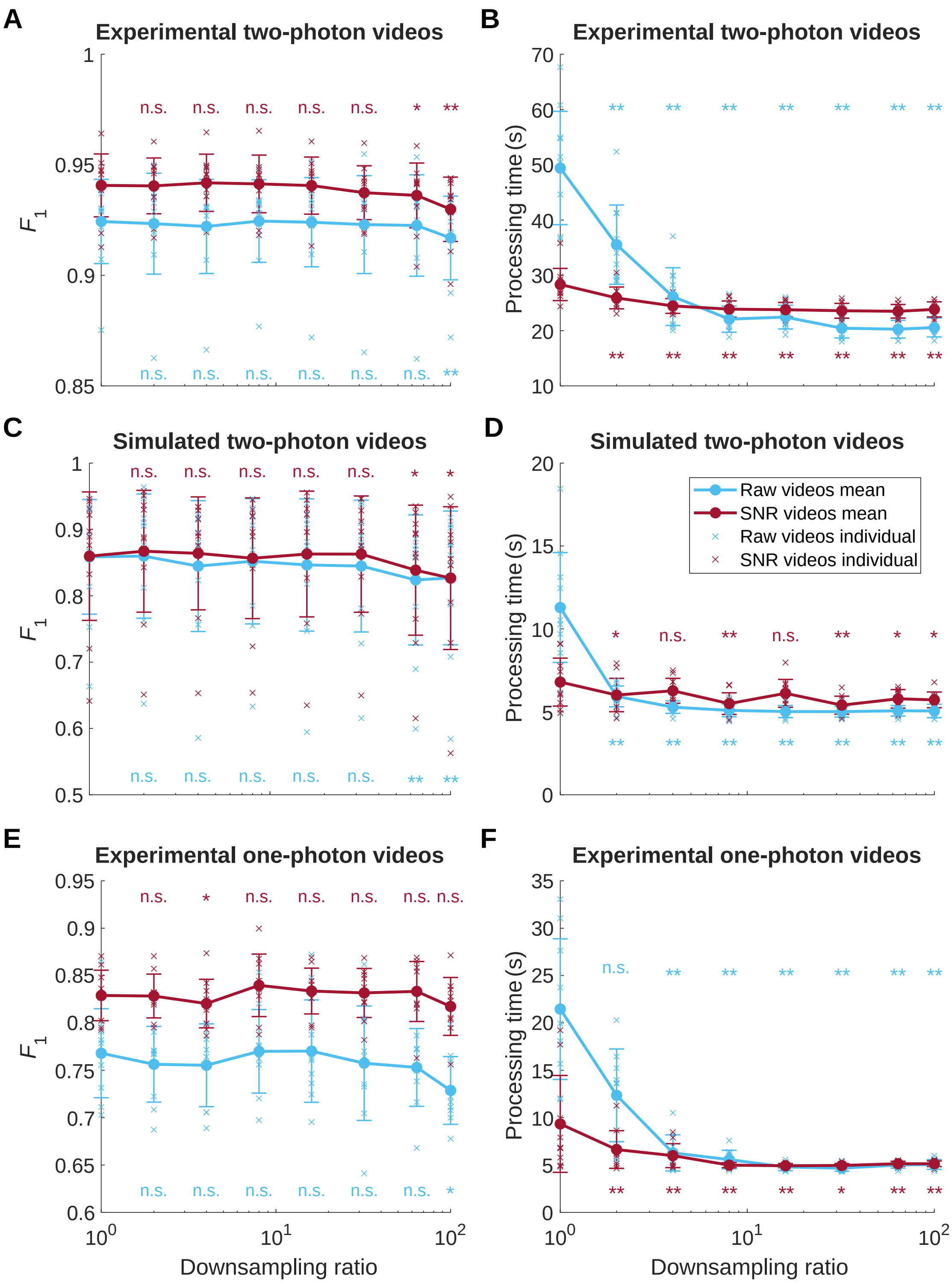

Supplementary Figure 5. Temporal downsampling can significantly improve the speed of TUnCaT without sacrificing accuracy.

(A, C, E) The F_1 scores of TUnCaT on **(A)** experimental two-photon videos, **(C)** simulated two-photon videos, and **(E)** experimental one-photon videos during cross-validation for raw and SNR videos using temporal downsampling with different downsampling ratios. The F_1 scores when using downsampling

ratios below 32 were generally not significantly different from the F_1 scores when not using downsampling. The labels above and below the curves show the significance of comparisons between F_1 scores when using downsampling and F_1 scores when not using downsampling (equivalent to downsampling ratio = 1) (*$p < 0.05$, **$p < 0.005$, n.s. - not significant, two-sided Wilcoxon signed-rank test; $n = 10$ videos for **(A)** and **(C)** and $n = 9$ videos for **(E)**; error bars are standard deviations).

(B, D, F) The processing times of TUnCaT on **(B)** experimental two-photon videos, **(D)** simulated two-photon videos, and **(F)** experimental one-photon videos for raw and SNR videos using temporal downsampling with different downsampling ratios. The processing time generally significantly decreased with increasing downsampling ratios, but asymptoted when the downsampling ratio was above 8. The labels above and below the curves show the significance of comparisons between the processing times when using downsampling to the processing times when not using downsampling (*$p < 0.05$, **$p < 0.005$, n.s. - not significant, two-sided Wilcoxon signed-rank test; $n = 10$ videos for **(B)** and **(D)** and $n = 9$ videos for **(F)**; error bars are standard deviations).

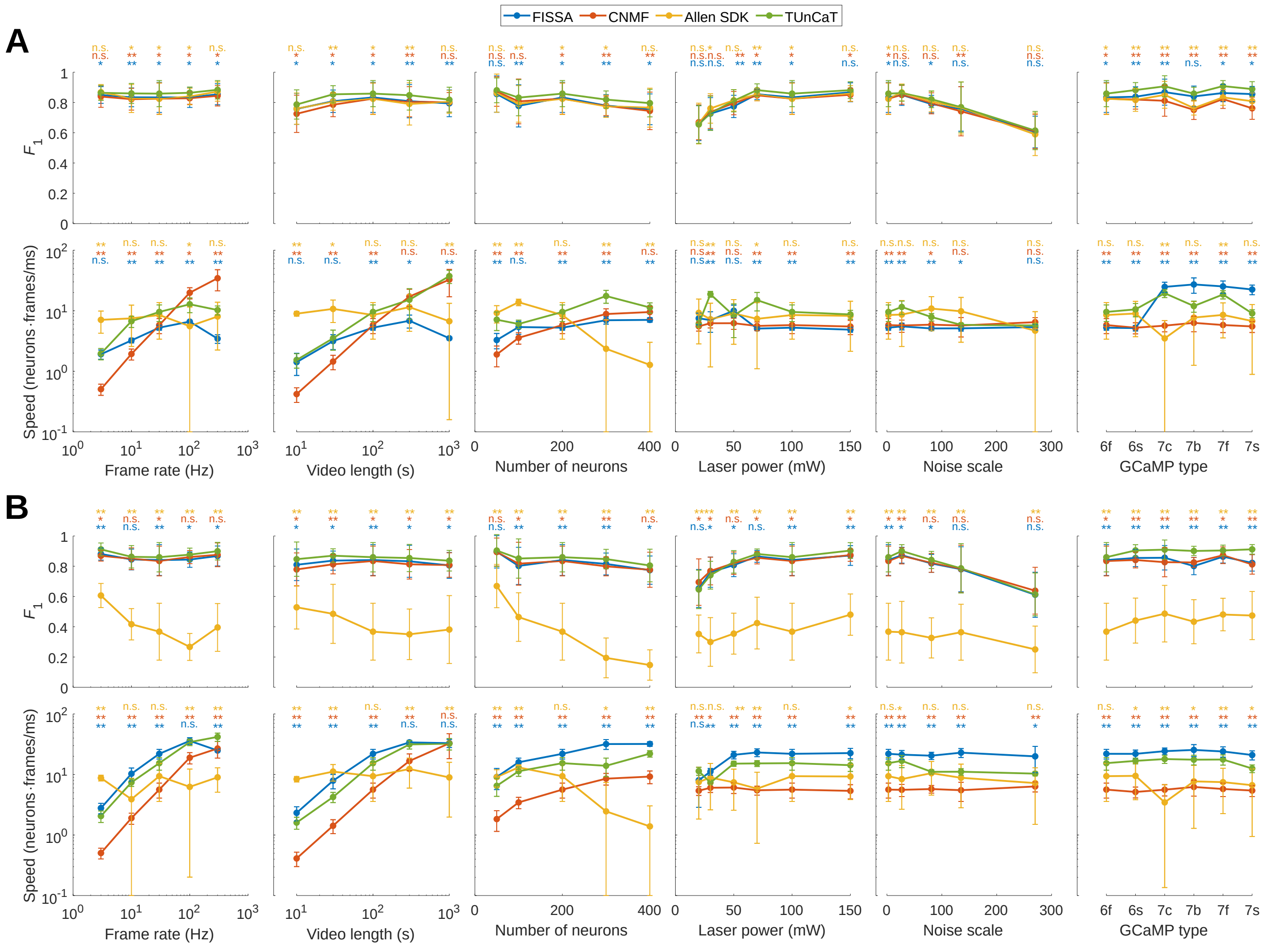

Supplementary Figure 6. TUnCaT was generally more accurate than peer algorithms when processing simulated two-photon videos with various simulation parameters and calcium sensors.

(A) *Top*: The F_1 scores of TUnCaT during 10-round cross-validation were generally superior to that of the other methods on raw videos. *Bottom*: The processing speeds (neurons · frames/s) were comparable to or faster than FISSA, CNMF, and the Allen SDK. The labels on top of the curves show the significance of the difference between the other methods and TUnCaT (*$p < 0.05$, **$p < 0.005$, n.s. - not significant, two-sided Wilcoxon signed-rank test, $n = 10$ videos; error bars are standard deviations). We simulated the videos with varying simulation parameters, including (from left to right) recording frame rate, video length, number of neurons, laser power, noise scale, and GCaMP type. We used the following values for these parameters when they were not varying: frequency = 30 Hz, processed video length = 100 s, number of neurons = 200, laser power = 100 mW, noise scale = 2.7, and calcium sensor = GCaMP6f. These parameters are typical in two-photon imaging experiments.

(B) Parallel to **(A)**, but for SNR videos.

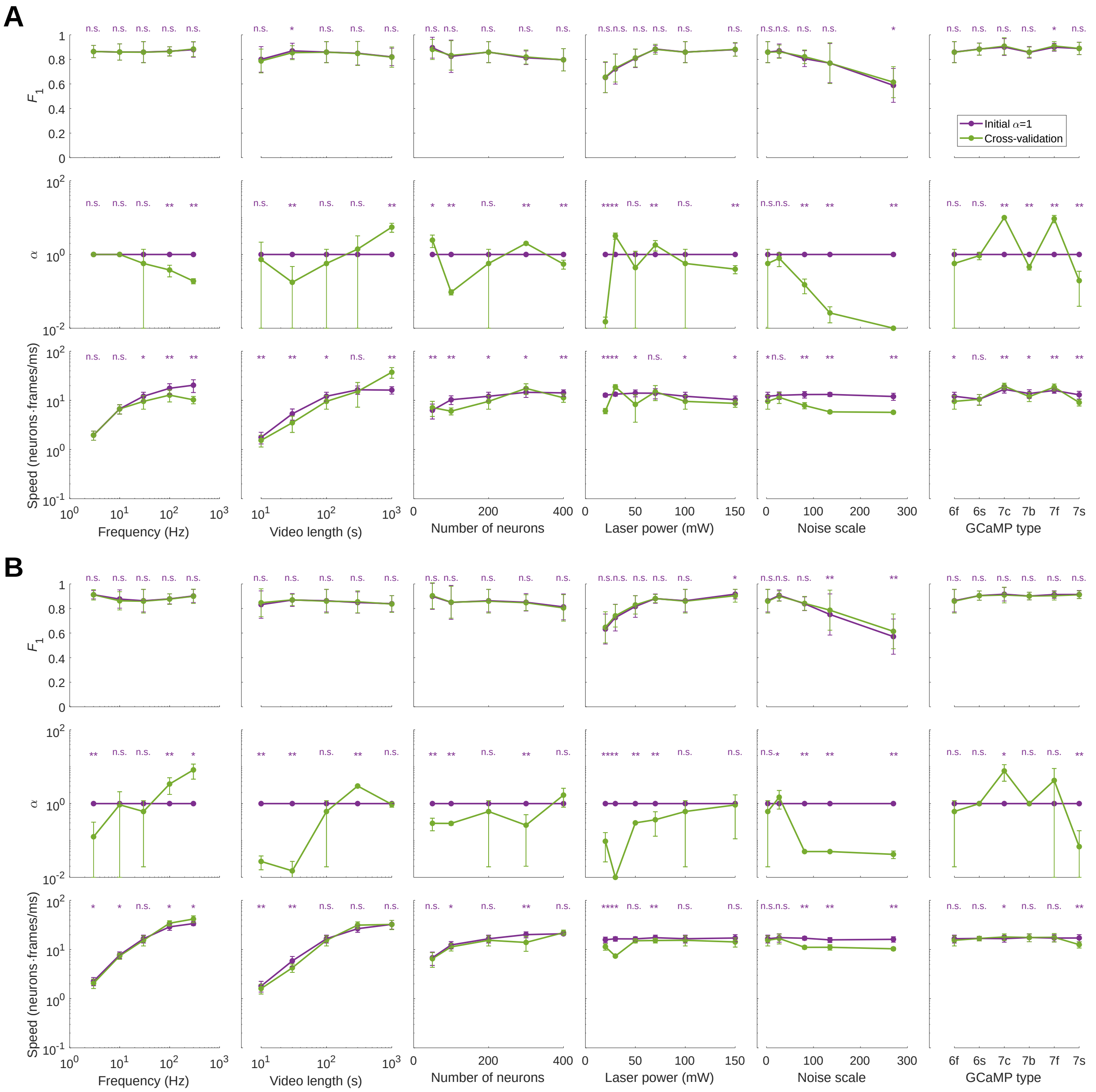

Supplementary Figure 7. Using α = 1 can achieve similar accuracy with using α optimized through cross-validation when processing simulated two-photon videos with various simulation parameters and calcium sensors.

(A) *Top*: The F_1 scores of TUnCaT during 10-round cross-validation when using a user-defined initial $\alpha = 1$ were very close to the F_1 scores when using α optimized through cross-validation, except on videos with very large noise scale. *Middle*: The optimized α through cross-validation, while a constant $\alpha = 1$ was also shown as a reference. *Bottom*: The processing speeds (neurons · frames/s) when using a user-defined initial $\alpha = 1$ were different from the speeds when using α optimized through cross-validation, because larger α resulted in faster processing speed. The labels on top of the curves show the significance of the difference between the two α selection strategies (*$p < 0.05$, **$p < 0.005$, n.s. - not significant, two-sided Wilcoxon signed-rank test, $n = 10$ videos; error bars are standard deviations).

We simulated the videos with varying simulation parameters, including (from left to right) recording frame rate, video length, number of neurons, laser power, noise scale, and GCaMP type. We used the following values for these parameters when they were not varying: frequency = 30 Hz, processed video length = 100 s, number of neurons = 200, laser power = 100 mW, noise scale = 2.7, and calcium sensor = GCaMP6f. These parameters are typical in two-photon imaging experiments.

(B) Parallel to **(A)**, but for SNR videos.
